# Antiretroviral APOBEC3 cytidine deaminases alter HIV-1 provirus integration site profiles

**DOI:** 10.1038/s41467-022-35379-y

**Published:** 2023-01-10

**Authors:** Hannah O. Ajoge, Tyler M. Renner, Kasandra Bélanger, Matthew Greig, Samar Dankar, Hinissan P. Kohio, Macon D. Coleman, Emmanuel Ndashimye, Eric J. Arts, Marc-André Langlois, Stephen D. Barr

**Affiliations:** 1 Western University, Schulich School of Medicine and Dentistry, Department of Microbiology and Immunology, London, ON Canada; 2 Department of Biochemistry, Microbiology and Immunology, Faculty of Medicine, University of Ottawa, Ottawa, ON Canada; 3 Ottawa Center for Infection, Immunity and Inflammation (CI3), Ottawa, ON Canada

**Keywords:** Retrovirus, Innate immunity, Restriction factors

## Abstract

APOBEC3 (A3) proteins are host-encoded deoxycytidine deaminases that provide an innate immune barrier to retroviral infection, notably against HIV-1. Low levels of deamination are believed to contribute to the genetic evolution of HIV-1, while intense catalytic activity of these proteins can induce catastrophic hypermutation in proviral DNA leading to near-total HIV-1 restriction. So far, little is known about how A3 cytosine deaminases might impact HIV-1 proviral DNA integration sites in human chromosomal DNA. Using a deep sequencing approach, we analyze the influence of catalytic active and inactive APOBEC3F and APOBEC3G on HIV-1 integration site selections. Here we show that DNA editing is detected at the extremities of the long terminal repeat regions of the virus. Both catalytic active and non-catalytic A3 mutants decrease insertions into gene coding sequences and increase integration sites into SINE elements, oncogenes and transcription-silencing non-B DNA features. Our data implicates A3 as a host factor influencing HIV-1 integration site selection and also promotes what appears to be a more latent expression profile.

## Abstract (web summary)

Antiretroviral APOBEC3 may contribute to HIV-1 latency. In this study, Ajoge and Renner et al. identify a previously undescribed function of human APOBEC3 proteins in redirecting integrations of HIV-1 DNA into more transcriptionally inactive regions of the genome.

## Introduction

The human A3 family is comprised of seven members, five of which have demonstrated biologically relevant antiviral activity against HIV-1: APOBEC3D (A3D), APOBEC3F (A3F), APOBEC3G (A3G), certain haplotypes of APOBEC3H (A3H), and one polymorphic variant of APOBE3C (A3C)^1–4^. When HIV-1 infects a new CD4+ monocyte or lymphocyte, A3 proteins associate with viral proteins and RNA, resulting in their encapsidation within nascent egressing virions^5^. Virion-packaged A3 then exert their antiretroviral activity in the target cells during reverse transcription primarily by deaminating cytosines (C) into uracils (U) in negative sense single-stranded viral DNA (vDNA) replication intermediates^6–8^.

Very high levels of deamination, called hypermutation, are observed early in the infection that thoroughly inactivate the virus^6^. However, HIV-1 can overcome the effects of A3 proteins by the increased expression of viral infectivity factor (Vif), which binds to and induces the polyubiquitination of the five anti-HIV-1 A3 proteins, thereby orchestrating their progressive depletion by proteasomal degradation^9–11^. Consequently, nascent egressing virions package decreasing amounts of A3 proteins until the proteins have been expunged from the cytosol by Vif^12^. These viruses devoid of A3, or even with highly reduced protein levels of the restriction factor, can freely infect new cells to help rapidly spread the infection. Retaining low rates of A3 mutagenesis, or hypomutation, are believed to be important contributors to the genetic evolution of HIV-1^13^.

A3 proteins can also restrict HIV-1 replication via mechanisms other than deamination (e.g., binding to the viral RNA or viral reverse transcriptase (RT), which reduces vDNA synthesis)^14–20^. It was previously shown that A3G and A3F can interact with the viral integrase (IN) and RT, but the role of this binding on viral integration is not yet clear^21–24^. More importantly, it was shown that A3F and A3G proteins can compromise viral integration efficiency by modifying or altering adequate processing of the extremities of the long terminal repeats (LTR) of the virus^25,26^. It is unknown how this may affect HIV-1 proviral integration site selection.

Upon synthesis of proviral DNA, a pre-integration complex (PIC) comprised of viral and host (e.g., A3) proteins translocates to the nucleus in preparation for integration^27,28^. Proviral DNA integration into open chromatin involves host Lens Epithelium-Derived Growth Factor (LEDGF/p75) binding to the viral IN and polyadenylation specificity factor 6 (CPSF6) at the LTR ends (i.e., the intasome)^29–32^. This HIV-1 intasome favors integration in chromatin that is bent and associated with histones, active transcription units, regions of high G/C content, high gene density, high CpG island density, high frequencies of short interspersed nuclear elements (SINEs) (e.g., Alu repeats), epigenetic modifications and specific nuclear regions such as close to nuclear pore complexes^32–35^. In addition, non-B DNA structures potentially influence HIV-1 integration site targeting^36^. At least 10 non-B DNA conformations exist including A-phased motifs, inverted repeats, direct repeats, cruciform DNA, guanine quadruplex (G4) DNA, slipped DNA, mirror repeats, short-tandem repeats, triplex repeats, and Z-DNA^37–40^.

While A3 proteins primarily act during reverse transcription to restrict HIV-1 through both deamination-dependent and independent mechanisms, A3F, and to a lesser extent A3G, remains associated with the PIC while it traffics into the nucleus^41^. In this study, we investigated the influence of A3 proteins on HIV-1 integration site selection. We found that both A3F and A3G have an important impact on integration site selection with both A3 deaminase-dependent and -independent activities contributing to this effect.

## Results

### A3F and A3G strongly inhibit HIV-1 infection and integration in a dose-dependent manner

**Fig. 1:**
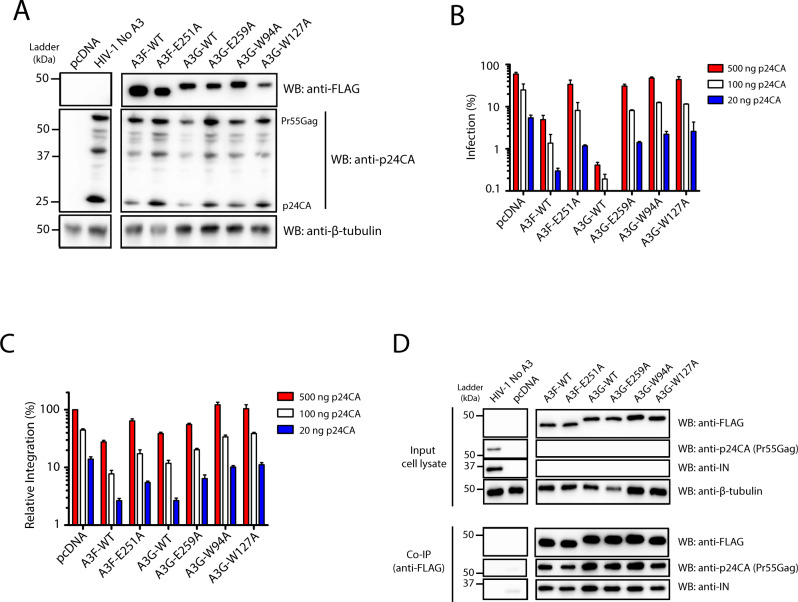
A3-mediated restriction of HIV-1 integration. **A** Western blot analysis of virus producer cells. HIV-1 pseudotyped virus was produced in 293 T cells by co-transfection of plasmids coding for NL4-3-ΔEnv/ΔVif/eGFP, VSV-G, and either empty pcDNA 3 plasmid (HIV-1 no A3), or each of the A3 expression plasmids. The Control lane is the transfection of the pcDNA in absence of virus. Cell lysates were subjected to SDS-PAGE and Western blot analysis. **B** CEM-SS cells were infected with the amounts of virus as indicated after normalization to capsid (p24CA) protein, as determined by ELISA. Infection was measured as the percentage of eGFP+ cells by flow cytometry. Data are presented as mean values ± SD. **C** Integrated provirus in the CEM-SS cells from **B** was quantified using Alu-based PCR combined with nested qPCR. Data are presented as mean values ± SD. **D** 293 T cells were transfected for the expression of each of the A3 proteins, HIV-1 (no A3), or pcDNA (cells transfected with pcDNA plasmid). Viral and cell lysates were mixed together and subjected to co-immunoprecipitation using an anti-FLAG antibody and analyzed by western blotting. Data shown are representative of three independent experiments. Source data are provided as a Source Data file.

Through the transfection of 293 T cells, we produced HIV-1 (NL4-3_ΔVif/ΔEnv-eGFP_)pseudotyped with vesicular stomatitis virus envelope glycoprotein (VSV-G) in the presence of wild-type (wt) or deamination-defective mutant forms of A3F [E251A] and A3G [E259A]. We also included the A3G nucleic acid-binding defective mutants A3G [W94A] and A3G [W127A]^14^. Within the non-catalytic N-terminal A3G domain, W94 is part of the SWSPCxxC zinc-coordinating motif while W127 is within ARLYYFW. These tryptophan residues are important for general nucleic acid-binding ability, substrate sequence recognition, and protein oligomerization^14,21,42–44^. While both W94A and W127A mutations diminish RNA binding, the W127A substitution is unique because it prevents the homodimerization of A3G which results in reduced processivity^14^. This highlights the importance of dimerization for the function of A3G catalytic activity in the presence of ssDNA^45^. Equal amounts of virus produced with each A3 were used to infect the permissive human T4-lymphoblastoid cell line CEM-SS. Productive infection of CEM-SS cells was determined 48 h post-infection by flow cytometry by way of virus-encoded eGFP reporter expression (Figs. 1A, S1). Alternatively, infected cells were harvested for genomic DNA (gDNA) extraction for the quantification of proviral integration levels and the downstream analysis of integration sites. Three different amounts of input virus were used for the infections in addition to producing virus in the presence of increasing amounts of A3 proteins (Figs. 1B, S1). Increasing the amount of A3 plasmid used for virus production had a noticeable impact on HIV-1 particle release (Fig. S2).

Potent restriction of HIV-1 was observed with wild-type (wt) A3F, and even more notably with wt A3G (Figs. 1B, S1). Both catalytically inactive A3F [E251A] and A3G [E259A] demonstrated significantly less restriction. Our group has previously established that A3G [W94A] and A3G [W127A] each have diminished restriction capabilities but remained capable of viral DNA editing^14^. These mutants had minor effects on the overall infection and integration efficiencies (Fig. 1B, C). Also, as expected, the level of inhibition by A3F and A3G were dependent on the amount of A3 proteins expressed during virus production (Fig. S1).

Given the multiple editing-dependent and -independent restriction mechanisms of A3, productive infection was correlated with overall levels of proviral integration. We measured relative integrated HIV-1 proviral DNA copy numbers by Alu-PCR followed by droplet digital PCR (ddPCR) targeting the HIV-1 LTR in one assay, the reporter eGFP gene as a control in another with input cellular DNA normalized to amplified actin DNA (Figs. 1C, S3)^46^. Integration levels tracked closely with infection levels, with the exception of the wt A3G and A3F proteins that exhibit proportionately more apparent restriction of infection than integration. This is unsurprising, as eGFP reporter expression and fluorescence relies on the genetic integrity of its coding sequence, which is frequently inactivated by A3F and A3G hypermutation^47^.

### A3F and A3G interact with the viral Gag and IN

Several reports have shown that A3F and A3G interact with HIV-1 Gag and with IN in an RNA-dependent manner, which are both components of the PIC^21–23,41,48,49^. However, binding to the A3 variants with inactive deaminase and defective nucleic acid-binding properties have not been previously assessed in parallel. For this study, it was essential to ascertain whether the various A3 proteins can interact directly or indirectly with IN as this interaction may be critical for PIC formation and integration site selection. To characterize HIV-1 IN and Gag interactions with the various A3 proteins, lysates of cells transfected with FLAG-tagged A3 variants or HIV-1 were co-incubated and then co-immunoprecipitated using anti-FLAG and analyzed by Western blotting using anti-IN and anti-p24CA. As shown in Fig. 1D, all A3 proteins, including the deaminase-inactive and nucleic acid-binding mutants co-immunoprecipitated with similar efficiencies with IN and Gag suggesting direct or indirect (i.e., through protein complexes) interactions with A3.

### A3F and A3G alter HIV-1 integration site targeting of genomic features

To identify HIV-1 integration sites, we amplified integration sites in genomic DNA (gDNA) isolated from cells infected with HIV-1 produced in the presence of the various A3 proteins. Integration site profiles were generated using the Barr Laboratory Integration Site Pipeline (BLISIP) as described^36,50^. BLISIP measures integration site enrichment in and near genomic features such as CpG islands, DNAseI hypersensitivity sites (DHS), endogenous retroviruses, heterochromatic DNA regions (e.g., lamina-associated domains (LADs) and satellite DNA), SINEs, long interspersed nuclear elements (LINEs), low complexity repeats (LCRs), oncogenes, genes, simple repeats, and transcription start sites (TSS). In addition, BLISIP measures enrichment in and near the non-B DNA features A-phased motifs, cruciform motifs, direct repeats, G4 motifs, inverted repeats, mirror repeats, short-tandem repeats, slipped motifs, triplex motifs, and Z-DNA motifs.

**Fig. 2:**
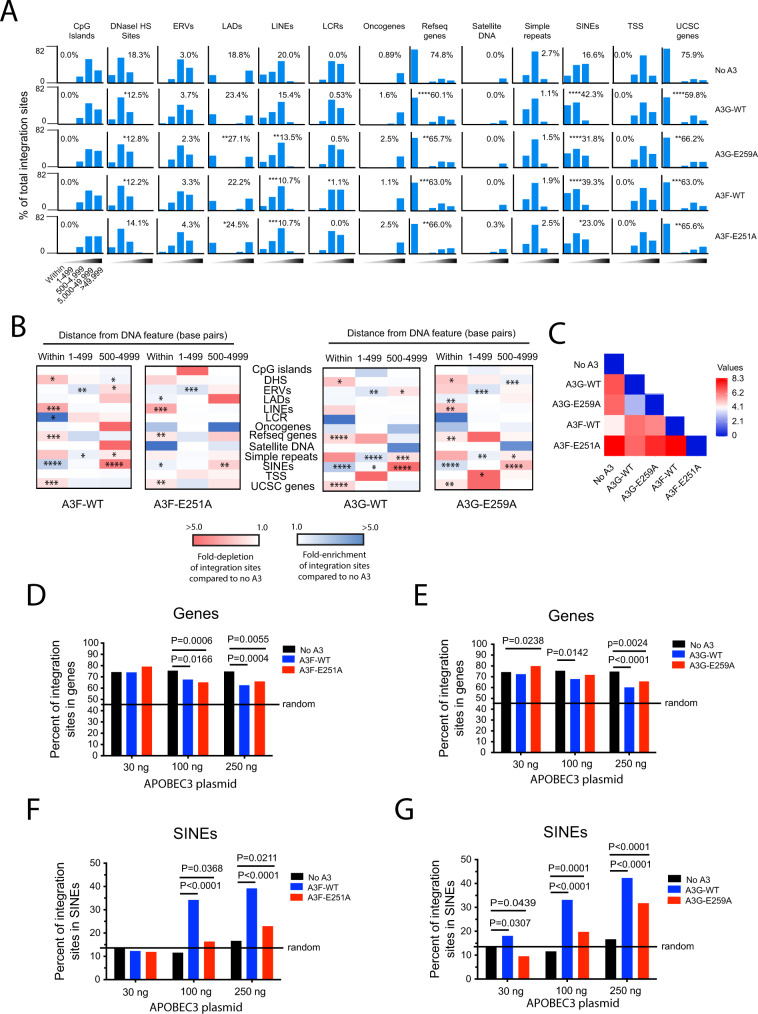
A3F and A3G expression alters integration site targeting of genomic features. **A** Frequency of integration sites within or at different distance intervals (1–499, 500–4999, 5000–49,999, or >49,999 bp) away from various common genomic features in CEM-SS T cells infected with HIV-1 generated in the presence of A3F-WT, A3F [E251A], A3G-WT, or A3G [E259A], or the absence of A3F or A3G (‘no A3’ control). Inset numbers refers to the percentage of total integration sites falling directly within the feature. The statistical comparison is with respect to the No A3 control. **B** Heatmaps depicting the fold-enrichment (blue shading) and depletion (red shading) of integration sites at various distance intervals compared to the ‘no A3’ control virus. **C** Pairwise distance matrix was used to determine the overall similarity between the integration site profiles of CEM-SS cells infected with either the no A3 control virus or A3F-WT, A3F [E251A], A3G-WT, or A3G [E259A] virus. The fold-enrichment and depletion values in each distance bin of each common DNA feature were used in the comparison. The heatmap shows the distance matrix calculated by Euclidean distance as the measurement method. Stronger relationships are indicated by the darker blue color and weaker relationships by darker red color. **D**, **E** Percentage of total integration sites located in genes for CEM-SS cells infected with A3F-WT, A3F [E251A], A3G-WT, or A3G [E259A] virus generated from cells expressing increasing concentrations of A3 protein. **F**, **G** Percentage of total integration sites located in SINEs for CEM-SS cells infected with A3F-WT, A3F [E251A], A3G-WT, or A3G [E259A] virus generated from cells expressing increasing concentrations of A3 protein. Shaded triangles represent the different distance bins with the darkest shading representing distances further away from the feature. *P < 0.05, **P < 0.01, ***P < 0.001, ****P < 0.0001; Fisher’s exact test, two-sided. Source data are provided as a Source Data file.

Target cells infected with viruses containing A3F or A3G exhibited a significant increase in integration in and near SINEs compared to viruses not containing A3F or A3G (39% and 42%, respectively) compared to 17%; *p* < 0.0001) (Fig. 2A, B, Supplementary Data File 1 and Supplementary Data File 2). Integration was also significantly enriched adjacent (1–500 nucleotides) to simple repeats for A3F and A3G in relation to the control with no A3 (26% and 35%, respectively) compared to 18%; *p* < 0.05). In addition, integration with A3F and A3G was modestly increased in and near ERVs, LADs, oncogenes and LCRs compared to the no A3 control. Notably, in the presence of A3F and A3G, integration was significantly decreased in genes compared to the no A3 control (63% and 60%, respectively) compared to 75%; *p* < 0.001). For comparison, 46% of integration sites occur in genes randomly (Supplementary Data File 3). Integration was also decreased in DHS and LINEs.

Using the A3F and A3G mutants lacking deaminase activity, there was a significant increase in integration in SINEs and a significant decrease in integration in genes, but not to the same magnitude observed with their wild-type counterparts. Similar to their wt counterparts, integration with A3F [E251A] and A3G [E259A] was modestly increased in or near ERVs, LADs and oncogenes (Fig. 2A, B, Supplementary Data File 1 and Supplementary Data File 2). To compare the overall similarity in integration site profiles between the various A3 constructs, we performed a pairwise analyses of the integration site profiles based on integration site enrichment or depletion within each of the bins ‘within,’ ‘1–499 bp’, and ‘500–4999 bp’, capturing all sites within 5000 bp of each genomic feature. As shown in Fig. 2C, the integration site profiles of the various A3-containing viruses differed from each other (*p* = 0.0398; two-way ANOVA, DF = 37), with A3G and A3G [E259A] sharing the most similarity in profiles.

Given that the largest differences in integration site selection were observed within genes and SINEs, we asked if these preferences were A3 dose-dependent. Indeed, increasing concentrations of A3 resulted in a decrease in the percentage of integrations sites in genes (Fig. 2D, E and Supplementary Data File 3). Conversely, increasing concentrations of A3 constructs resulted in a dose-dependent increase in the percentage of integrations sites in SINEs (Fig. 2F, G and Supplementary Data File 3). Together, these data show that A3F and A3G influence HIV-1 integration site targeting and that the deamination activity of A3F, and to a lesser extent A3G, influences the magnitude of this targeting.

### A3F and A3G expression alters integration site targeting of non-B DNA motifs

**Fig. 3:**
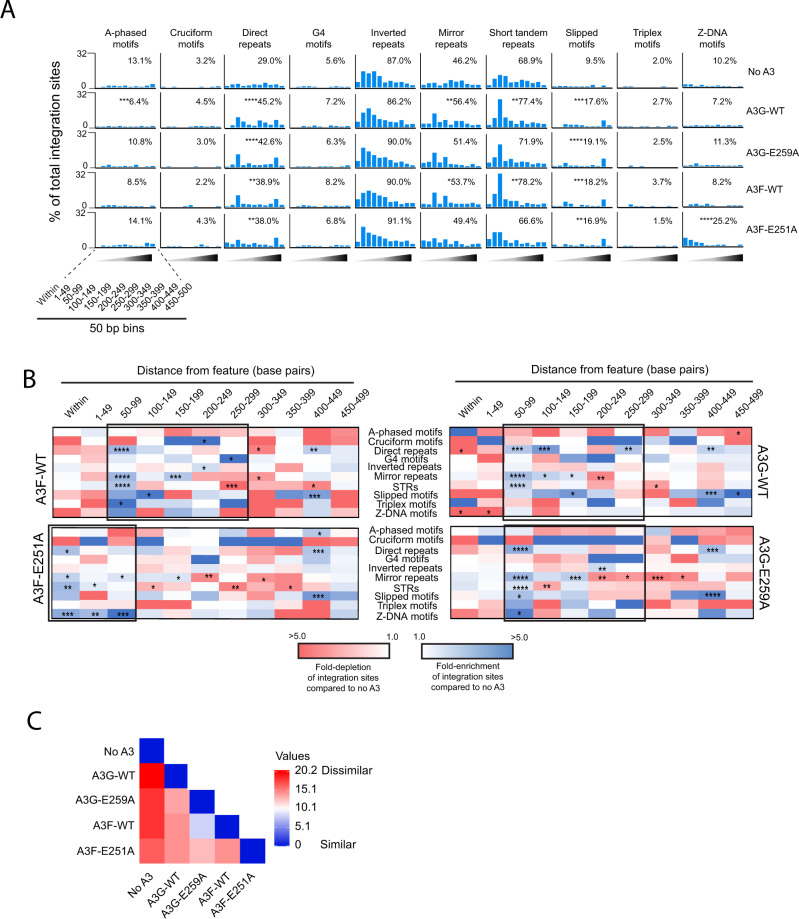
A3F and A3G alters integration site targeting of non-B DNA. **A** Frequency of integration sites within or in different 50 bp distance intervals (1–500 bp) away from various non-B DNA features in CEM-SS T cells infected with HIV-1 generated in the presence of A3F-WT, A3F [E251A], A3G-WT, or A3G [E259A], or the absence of A3G or A3F (‘no A3’ control). The inset numbers refer to the percentage of total integration sites falling within 500 bp of the feature. The statistical comparison is with respect to ‘no A3’. **B** Heatmaps depicting the fold-enrichment (blue shading) and depletion (red shading) of integration sites at various distance intervals compared to the ‘no A3’ control virus. Black boxes highlight regions of notable enrichment. **C** Pairwise distance matrix was used to determine the overall similarity between the different integration site profiles. The fold-enrichment and depletion values in each distance bin for each non-B DNA feature were used in the comparison. The heatmap shows the distance matrix calculated by Euclidean distance as the measurement method. Stronger relationships are indicated by the darker blue color and weaker relationships by darker red color. Shaded triangles represent the different distance bins with the darkest shading representing distances further away from the feature. *P < 0.05, **P < 0.01, ***P < 0.001, ****P < 0.0001; Fisher’s exact test, two-sided.

We then determined the impact of A3 on targeting non-B DNA motifs for integration. Cells infected in the presence of A3F or A3G exhibited enriched integration within 500 bp of most non-B DNA features (Fig. 3A and Supplementary Data File 4). Cells infected in the presence of A3F [E251A] or A3G [E259A] exhibited a similar level of integration near most non-B DNA compared to the control, except for direct repeats and slipped motifs, where a significant increase in integration was observed. Notably, A3F [E251A] exhibited a large increase in integration near Z-DNA compared to the other A3 constructs and the control (25% compared to 10% for the control; *p* < 0.0001).

To determine if there were differences in the distribution of integration sites near to the non-B DNA features, we compared the number of integration sites in bins of 50 bp up to 500 bp away from each non-B DNA motif (Fig. 3B and Supplementary Data File 5). Integration preferences of viruses produced with A3F, A3G, and A3G [E259A] clustered in a region 50–300 bp away from the non-B DNA. A3F [E251A] differed from the others in that integration clustered in a region within 100 bp of the features. Pairwise analysis of the integration site profiles (within 500 bp of the features) showed that while A3F and A3G [E259A] shared a surprising amount of similarity, all other integration profiles are different (*p* < 0.0001; two-way ANOVA, DF = 109) (Fig. 3C). Together, these data show that A3F and A3G influence HIV-1 integration site targeting of non-B DNA features with a substantial contribution of their deamination activities in the targeting of A-phased, mirror repeats, STRs, and Z-DNA features.

.

### A3G residues W94 and W127 impact HIV-1 integration site targeting

**Fig. 4:**
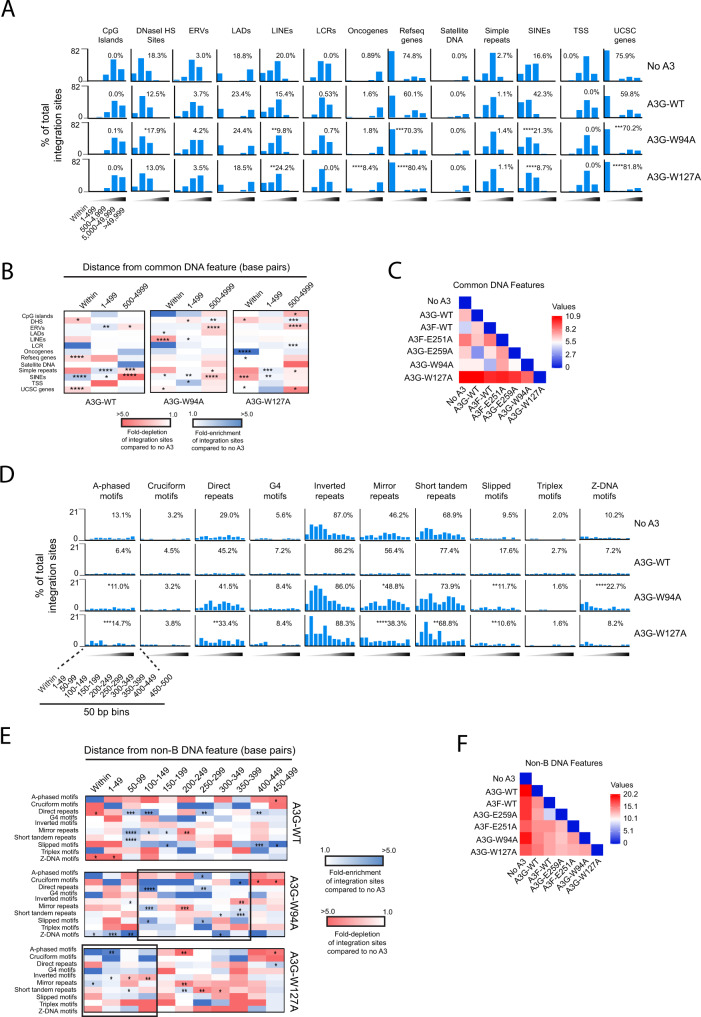
A3 residues W94 and W127 play a role in integration site targeting. **A** Frequency of integration sites within or at different distance intervals away from various common genomic features in CEM-SS T cells infected with HIV-1 generated in the presence of A3G-WT, A3G [W94A], A3G [W127A], or the absence of A3F or A3G (‘no A3’ control). The inset numbers refer to the percentage of total integration sites falling directly within the feature. The statistical comparison is with respect to A3G-WT. **B** Heatmaps depicting the fold-enrichment (blue shading) and depletion (red shading) of integration sites at various distance intervals compared to the ‘no A3’ control virus. **C** Pairwise distance matrix was used to determine the overall similarity between the integration site profiles of CEM-SS cells infected with either the no A3 control virus or A3F-WT, A3F [E251A], A3G-WT, A3G [E259A], A3G [W94A], or A3G [W127A] virus. The fold-enrichment and depletion values in each distance bin of each common DNA feature were used in the comparison. The heatmap shows the distance matrix calculated by Euclidean distance as the measurement method. Stronger relationships are indicated by the darker blue color and weaker relationships by darker red. **D** Frequency of integration sites within or at different distance intervals away from various non-B DNA features. The inset numbers refer to the percentage of total integration sites falling within 500 bp of the feature. The statistical comparison is with respect to A3G-WT. **E** Heatmaps depicting the fold-enrichment (blue shading) and depletion (red shading) of integration sites at various distance intervals compared to the ‘no A3’ control virus. Black boxes highlight regions of notable enrichment. **F** Pairwise distance matrix was used to determine the overall similarity between the integration site profiles. Shaded triangles represent the different distance bins with the darkest shading representing distances further away from the feature. *P < 0.05, **P < 0.01, ***P < 0.001, ****P < 0.0001; Fisher’s exact test, two-sided. Source data are provided as a Source Data file.

We analyzed the integration site profiles of cells infected with virus produced in the presence of A3G [W94A] or A3G [W127A] mutants to determine if these residues impact the ability of wt A3G to influence integration site targeting. Compared to their wt counterpart, A3G [W94A] and A3G [W127A] exhibited a significant increase in integration in genes and decreased integration in SINEs (Fig. 4A, B, and Supplementary Data File 1 and Supplementary Data File 2). In addition, A3G [W127A] exhibited a notable increase of integration events in oncogenes compared to wt A3G. Interestingly, while A3G [W94A] exhibited an intermediate phenotype between the control and wt A3G, A3G [W127A] seemed to exacerbate the integration site preferences of HIV-1. Pairwise analyses of all A3 integration site profiles (within 5000 bp of the various features) showed that A3G [W94A] was most similar to wt A3F and that A3G [W127] differed from all A3 variants tested (*P* = 0.014; two-way ANOVA, DF = 37) (Fig. 4C).

With respect to non-B DNA features, cells infected with A3G [W94A]- or A3G [W127A]-containing virus exhibited a significant increase in integration near A-phased motifs and decreased integration near mirror repeats and slipped motifs compared to wt A3G (Fig. 4D and Supplementary Data File 4). In addition, A3G [W127A] exhibited a significant increase in integration near Z-DNA. The distribution of integration sites in bins of 50 bp up to 500 bp away from each non-B DNA motif was similar between wt A3G and A3G [W94A], where sites clustered predominantly in a region 100–400 bp away from the features (Fig. 4E and Supplementary Data File 5). An exception was Z-DNA where sites were highly enriched in and within 100 bp of Z-DNA motifs. In contrast, integration sites from cells generated in the presence of A3G [W127A] tended to cluster within 150 bp of non-B DNA. Pairwise analysis of the integration site profiles (within 500 bp of the features) showed that while A3F and A3G [E259A] shared similarity, all other integration profiles are different (*p* < 0.0001; two-way ANOVA, DF = 109) (Fig. 4F). Together, these data show that A3G residues W94, and to a greater extent W127, differentially impact the ability of A3G to influence integration site targeting.

### A3F and A3G reduce the number of hotspots and clustering of integration sites

**Fig. 5:**
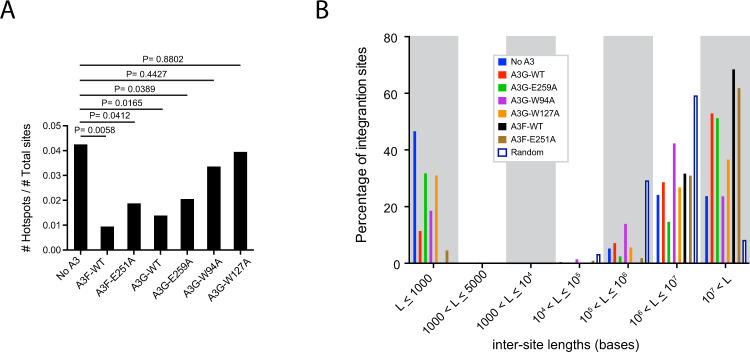
A3F and A3G reduce the number of integration hotspots and clustering of sites. **A** Analysis of integration site hotspots. A hotspot was defined as a 1 kb window in the genome hosting 4 or more unique integration sites. Integration hotspots are shown as a proportion of total integration sites from CEM-SS cells infected with HIV-1 produced in the presence of no A3F or A3G (“No A3”) control (blue bars), or from cells expressing A3F-WT (black bars), A3F [E251A] (brown bars), A3G-WT (red bars), A3G [E259A] (green bars), A3G [W94A] (purple bars), or A3G [W127A] (yellow bars). **B** Integration site clustering was assessed by comparing the spacing between integration sites genome-wide to the same number of uniformly distributed (random) sites. Distances between sites are collected in seven length (L) ‘bins,’ with the shortest intersite lengths to the left and the longest to the right. A matched random control dataset was generated in silico (see methods for details). Fisher’s exact test, two-sided.

The concept of an HIV-1 integration “hotspot” was introduced to describe areas of the genome where integrations accumulate more than expected by chance in the absence of any selection process^51^. Given our findings that A3F and A3G influence integration site targeting, we asked if they also impact the number of integration hotspots and clustering of sites. We defined an integration hotspot as a 1 kilobase (kb) gDNA fragment containing four or more unique integration sites. CEM-SS T cells infected with HIV-1 produced in the presence of A3F or A3G exhibited a substantial reduction in the number of hotspots compared to cells expressing no A3 (*p* < 0.05, Fisher’s exact test) (Fig. 5A and Supplementary Data File 6). A3F [E251A] and A3G [E259A] also exhibited a reduced number of hotspots indicating that the A3 deamination activities were not essential for this effect. In contrast, the presence of A3G [W94A] or A3G [W127A] exhibited no significant reduction in the number of integration site hotspots.

To document clustering of integration sites within genomic regions, we compared the distance between proviral integration sites (Fig. 5B). The control population of integration sites contained more short intersegment distances than expected by chance (i.e., random distribution), indicative of clustering. Viruses produced with A3F or A3G exhibited a striking reduction in clustering compared to the control cells (*p* < 0.0001, Fisher’s exact test). This reduction was lost in virus containing A3G [E259A] and A3G [W127A], and to a lesser extent A3G [W94A] and A3F [E251A]. Together, these data show that expression of A3F and A3G reduce the number of integration hotspots and clustering of integration sites.

### G-to-A mutations in the LTR alter integration site targeting in vitro and in vivo

**Fig. 6:**
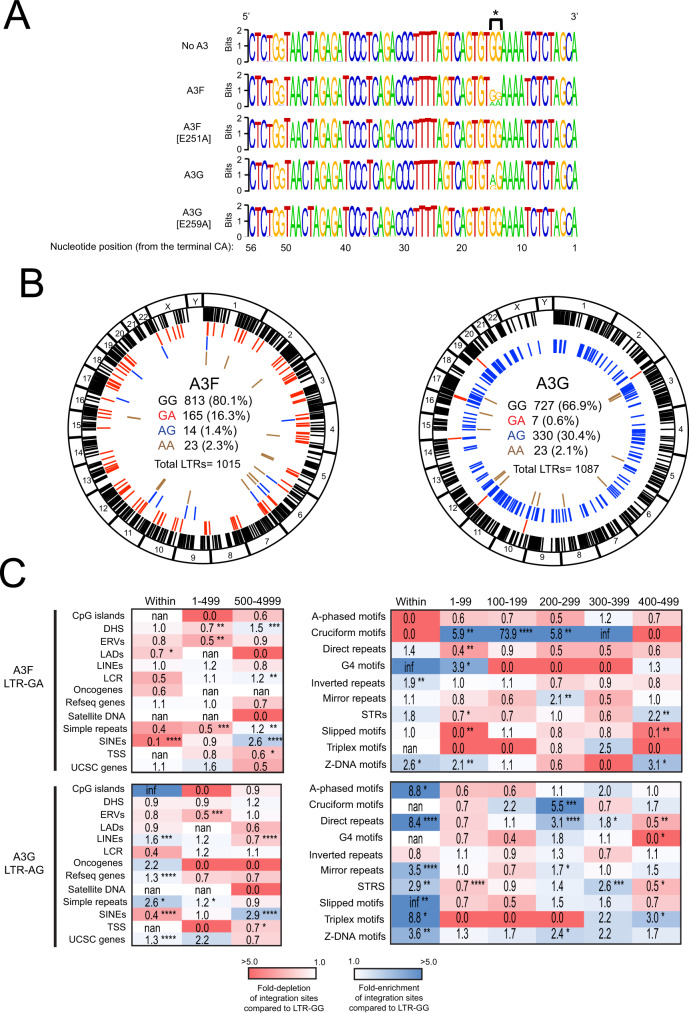
G-to-A mutations in the 3’ LTR alters integration site targeting in vitro. **A** LOGO representations of the terminal 56 nucleotides (A, green; C, blue; G, orange; T, red) of the 3’ LTRs of all integrated HIV-1 proviruses generated in the presence of either A3F, A3F [E251A], A3G, or A3G [E259A]. **B** Circa plots showing the integration sites of A3F- (left) or A3G- (right) containing viruses in the genome of infected CEM-SS cells. The outer ring represents the different chromosomes. The chromosomal locations of integration sites of proviruses containing GG (black), GA (red), AG (blue), and AA (brown) at positions 14 and 15 nucleotides from the LTR end are represented as colored ticks. The number and percentage of total sites are shown inside the circa plots. **C** Heatmaps depicting the fold-enrichment (blue shading) and depletion (red shading) of integration sites at various distance intervals from common genomic features (left) and non-B DNA features (right). Integration sites from A3F-LTR-GA (top) and A3G-LTR-AG (bottom) proviruses are shown. Fold changes are with respect to A3F-LTR-GG and A3G-LTR-GG, respectively. *P < 0.05, **P < 0.01, ***P < 0.001, ****P < 0.0001; Fisher’s exact test, two-sided. Infinite number (inf) represents 1 or more integrations were observed when 0 integrations were expected by chance. Not a number (nan) represents 0 integrations were observed and 0 were expected by chance.

To determine if deamination of LTR ends impacted integration site targeting, we aligned unique integrated 3’ LTR nucleotide sequences and represented them graphically as sequence logos using WebLogo^52,53^ (Fig. 6A). As expected, the 3’ LTR ends of the control and the deamination-defective A3 mutants A3F [E251A] and A3G [E259A] were highly similar. The 3’ LTR ends of A3F and A3G were also highly similar with the exception of the 2 nucleotides located at positions 14 and 15 from the end of the LTR. In the presence of A3F, 80.0% of the LTRs contained GG at these positions, 16.3% contained GA, 1.4% contained AG, and 2.3% contained AA (Fig. 6B). In the presence of A3G, 66.9% of the LTRs contained GG at these positions, 0.6% contained GA, 30.4% contained AG, and 2.1% contained AA.

To determine if G-to-A mutations at positions 14 and/or 15 from the 3’ LTR end correlated with an altered integration site profile, we compared the A3F and A3G integration site profiles of proviruses containing either GA or AG (respectively) to that with GG at positions 14 and 15 from the 3’ LTR end. A3F LTRs containing the GA dinucleotide (A3F-LTR-GA) exhibited a notable reduction in integration sites within SINEs and increased integration more distal (500–5000 bp) to SINEs (Fig. 6C, Supplementary Data File 7 and Supplementary Data File 8). Increased integration was also observed more distal to DHS, LCR, and simple repeats. Strikingly, the integration site profile changed dramatically with respect to non-B DNA features. A3F-LTR-GA sites were highly enriched (up to 74-fold) 1–400 bp from cruciform motifs (Fig. 6C and Supplementary Data File 7). Additionally, sites were enriched in and near G4 DNA, inverted repeats and Z-DNA. A3G LTRs containing the AG dinucleotide (A3G-LTR-AG) also exhibited reduced integration in SINEs and increased integration more distal to SINEs; however, unlike A3F-LTR-GA, integration was also increased in genes and simple repeats (Fig. 6C and Supplementary Data File 8). Similar to A3F-LTR-GA, A3G-LTR-AG exhibited a striking change in integration site profile with respect to non-B DNA. Integration was enriched in and/or near most non-B DNA features. Together, these data show that A3-induced G-to-A mutations at position 14 or 15 from the end of the 3’ LTR correlates with a significantly altered integration site profile with enrichment near transcription-silencing non-B DNA features.

**Fig. 7:**
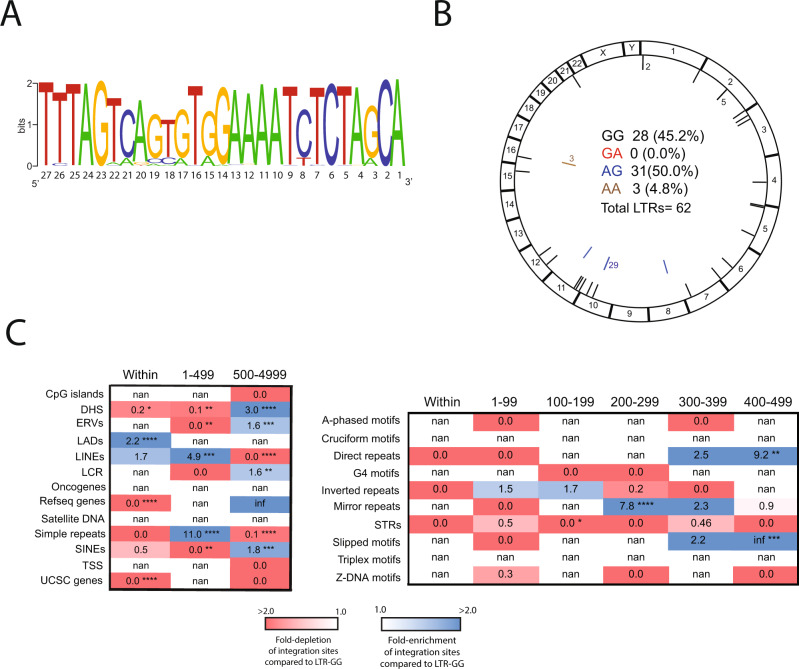
G-to-A mutations in the 3’ LTR alters integration site targeting in vivo. **A** LOGO representations of the terminal 27 nucleotides (A, green; C, blue; G, orange; T, red) of the 3’ LTRs of integrated HIV-1 proviruses in HIV-1 infected individuals. **B** Circa plot showing the integration sites of proviruses in the genome of infected individuals. The outer ring represents the different chromosomes. The chromosomal locations of integration sites of proviruses containing GG (black), GA (red), AG (blue), and AA (brown) at positions 14 and 15 nucleotides from the LTR end are represented as colored ticks. Numbers adjacent to ticks show the number of sites in that region that could not be distinguished by multiple ticks. The number and percentage of total sites are shown inside the circa plots. **C** Heatmaps depicting the fold-enrichment (blue shading) and depletion (red shading) of integration sites at various distance intervals from common genomic features (left) and non-B DNA features (right). Integration sites from proviral LTR-AG is shown and the fold changes are with respect to proviral LTR-GG integration sites. *P < 0.05, **P < 0.01, ***P < 0.001, ****P < 0.0001; Fisher’s exact test, two-sided. Infinite number (inf) represents 1 or more integrations were observed when 0 integrations were expected by chance. Not a number (nan) represents 0 integrations were observed, and 0 were expected by chance.

To determine if similar G-to-A mutations occur at positions 14 and 15 from the end of proviral LTR sequences from HIV-1 infected individuals, genomic DNA was isolated from peripheral blood mononuclear cells (PBMCs) from a cohort of 93 patients and used to sequence proviral LTRs and generate integration site libraries. We aligned unique integrated 3’ LTR nucleotide sequences identified and filtered as described for the aforementioned in vitro analysis and represented them graphically as sequence logos using WebLogo^52,53^ (Fig. 7A). Sequence analysis showed G-to-A mutations at positions 14 and 15 from the 3’ LTR end in similar proportions as that observed with A3G (Fig. 7A). Forty-five percent of the proviral LTRs contained the GG dinucleotide (LTR-GG), 50% contained AG (LTR-AG), 0% contained GA (LTR-GA), and 5% contained AA (LTR-AA) (Fig. 7B). Although the total number of integration sites for the LTR-GG and LTR-AG proviruses was low (48 sites total), likely due to the patients being on antiretroviral therapy, a comparison of the integration site profiles of proviruses containing LTR-AG (25 sites) versus LTR-GG (23 sites) showed a significant reduction in integration sites within genes and a significant increase in integration in lamina-associated domains (LADs). Similar to A3G-LTR-AG, integration sites from patient LTRs containing LTR-AG were enriched near several non-B DNA features, particularly repeat and slipped motifs, which are known to negatively impact gene expression (Fig. 7C and Supplementary Data File 9).

### A3F and A3G promote latent infections in vitro

**Fig. 8:**
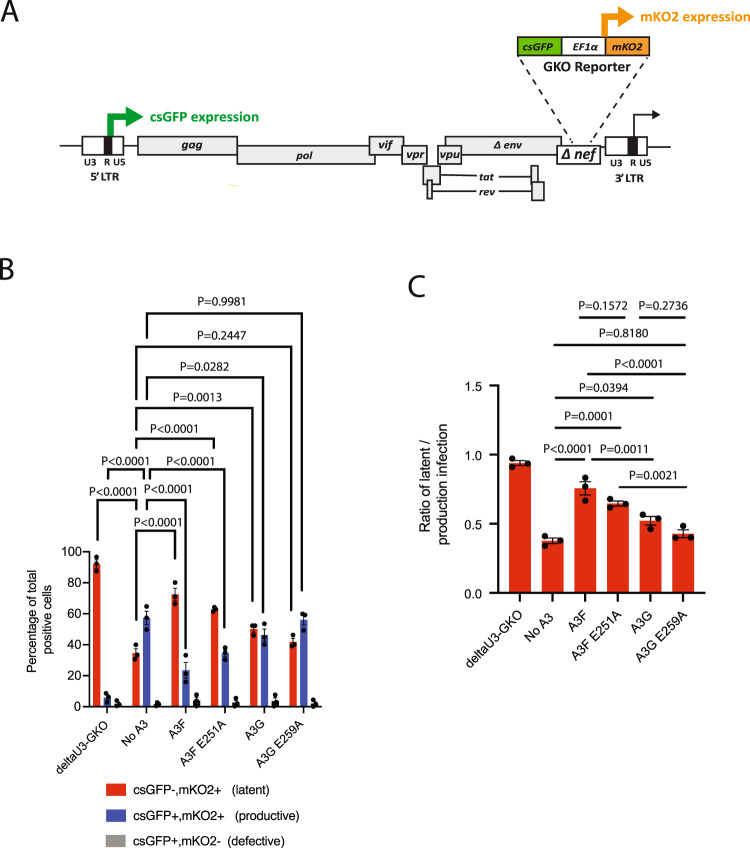
A3F and A3G promote latent infections in vitro. **A** Schematic of the HIV-1 GKO reporter vector showing the csGFP gene (green box) under transcriptional control of the HIV-1 5’ LTR promoter and the mKO2 gene (orange box) under control of the constitutive EF1alpha promoter. **B** CEM-SS cells were infected with HIV_GKO_ or HIV_GKO-ΔU3LTR_ in the presence or absence of A3F, A3F[E251A], A3G, or A3G[E259A] for 48 h. Using flow cytometry, live cells were gated on using Zombie NIR^TM^ staining and the percentage of double-positive (csGFP+, mKO2+) (blue bars) and single-positive cells ((csGFP−, mKO2+) (red bars) or (csGFP+, mKO2−) (gray bars)) is shown. Statistical analysis was performed using two-way ANOVA with Dunnett’s multiple comparisons test (degrees of freedom = 36). **C** The average proportion of latently infected cells (csGFP−, mKO2+) from panel **B** is shown. Statistical analysis was performed using one-way ANOVA with Tukey’s multiple comparisons test (degrees of freedom = 12). Data shown represents the mean values (±S.E.M.) from three independent experiments. Source data are provided as a Source Data file.

Given our finding that A3 promotes integration near transcription-silencing genomic features, we next determined if A3 expression alters the proportion of latently and productively infected cells. In these experiments, we utilized a dual-fluorescence HIV-1 reporter virus (HIV_GKO_) designed for the quantification of latently infected cells by flow cytometry (Fig. 8A)^54^. The HIV_GKO_ construct contains codon-switched enhanced green fluorescent protein (csGFP) under the transcriptional control of the 5’ LTR promoter and a distinct unrelated fluorescent protein mKO2 under control of an internal EF1α promoter. Productive infection of cells with HIV_GKO_ results in cells expressing both csGFP and mKO2 (csGFP+, mKO2+), whereas latent infection of cells results in only mKO2 expression (csGFP−, mKO2+). Cells exhibiting only csGFP expression (GFP+, mKO2−) were considered having a defective provirus integrated. Cells not expressing either marker (csGFP−, mKO2−) are considered either uninfected, and/or containing proviruses latent for both csGFP and mKO2 expression. These (csGFP−, mKO2−) cells were excluded from the analysis. A representative flow cytometry experiment is presented in Fig. S4.

After infection of CEM-SS cells with HIV_GKO_ in the absence of A3, we observe a higher proportion of cells with productive integrations (csGFP+, mKO2+) compared to latent integrations (csGFP−, mKO2+) (Fig. 8B, C). In contrast, cells infected with an equivalent amount of HIV_GKO_ in the presence of A3F or A3F[E251A] resulted in a significantly higher proportion of latent integrations compared to productive integrations. HIV_GKO_ in the presence of A3G also resulted in a significant increase in latent integrations but to a lesser extent than with A3F and A3F[E251A]. No significant changes in the proportion of latent or productive integrations were observed in the presence of A3G[E259A] compared to the no A3 control. As expected, the negative control HIV_GKO_ virus lacking the U3 promoter region of the 3′ LTR (HIV_ΔU3-GKO_) resulted in an integrated virus expressing mKO2 only (csGFP−, mKO2+). Together, these data show that A3F and A3G significantly increase the proportion of latent integrations in vitro and that the deamination activities of A3G and A3F contribute to this increase.

## Discussion

Despite the presence of numerous cellular host restriction factors that collaboratively work to inhibit early stages of HIV-1 infection, integration of the HIV-1 genome into the host genome can still occur. An integration event will have varying outcomes depending on the genomic site of integration. These may involve direct modulation of host gene networks, controlling levels of viral transcription, and in some cases influence the outcome of active or latent infection^55,56^. Normally, A3 proteins play a major role in hindering reverse transcription of the HIV-1 RNA genome and mutating replication intermediates; however, HIV-1 Vif circumvents this restriction by reducing A3 protein levels in productively infected cells. Additionally, some A3 proteins including A3F and A3G have been shown to interact with the PIC and are translocated into the nucleus^41^. The consequences of this localization in the nucleus and its impact on HIV-1 integration was previously unknown. Here we have shown that A3F and A3G significantly alter HIV-1 integration site selection (Fig. S5).

HIV-1 has an integration site preference for actively transcribing genic regions, particularly those activated during the infection^57,58^. The selection process undoubtedly plays an important role in expansion and persistence of infected cells, as was demonstrated in patients on cART^59^. Host cellular proteins are known to play critical roles in HIV-1 integration site selection. For example, LEDGF/p75 and CPSF6 promote integration into actively transcribing genes residing in gene-dense regions^29,30,32,58,60–64^. Here we showed that host cellular A3 expression shifts the accumulation of HIV-1 integration sites away from genes and towards SINEs in a dose-dependent manner. Moreover, increased A3 expression reduced the number of integration site hotspots in the human genome suggesting that A3 increases integration site diversity. Given the ability of A3 to interact with integrase within the PIC, it will be interesting to learn if A3 sterically interferes with the ability of integration site targeting factors such as LEDGF and CPSF6 to bind and target the PIC to transcriptionally active regions of the nucleus and genome. Similarly, deamination activity of A3 on the 3’ LTR (e.g., positions 14/15) could alter protein-nucleic acid interactions within the PIC or between the PIC and integration site targeting factors. Of course, during later rounds of HIV-1 replication, A3 levels are reduced by Vif, which could be another mechanism by which the virus promotes integration into more active regions of the genome to help establish productive infection. Given this temporal gradient of A3 expression, their effects on integration likely occur at the earlier moments after a new cell is infected when it releases permissive levels of A3 in HIV virions. Additionally, as shown herein and by other groups, there is evidence that infectious deaminated proviral genomes do exist in infected individuals, and therefore support an opportunity for A3 to influence integration^65–68^. While most of these deaminated proviral genomes are defective in some manner, some can still produce HIV RNA and viral proteins and thereby contribute to chronic immune activation in the absence of infectious particle egress^56^. Additionally, our experimental data support that A3 proteins can promote latent integrations. We define these in this experimental system as integrated proviruses where the 5’LTR promoter fails to express the GFP reporter under its control but maintains mKO2 reporter expression from an internal promoter. This is observed for both catalytically active and inactive A3 variants, further providing support that a simple physical association of the deaminases with the PIC may influence the expression outcome of integrated HIV proviral DNA. Since integration sites also play a critical role in the expansion and persistence of HIV-1-infected cells, these A3-directed integration sites could have a role in the persistence of latent infection in patients. Finally, inactivating mutations from the catalytic activities of the wt A3 proteins may also contribute to the latent phenotype. More work on human samples from HIV patients is needed to determine if long-lived latently infected cells predominantly harbor the mutational signatures at positions 14 and 15 in the LTR that we identified here.

The ability of A3 to alter the integration site profile was partially dependent upon the deaminase activity of both A3F and A3G, but more strongly dependent upon the nucleic acid-binding ability of A3G. We previously showed that the nucleic acid-binding mutants A3G [W94A] and A3G [W127A] are encapsidated into virus particles, albeit to a reduced degree, and exhibited similar deaminase activity compared to wt A3G^14^. In addition, these mutants did not reduce late reverse transcripts or integration of the virus^14^. Our finding that these same mutants displayed unique influences on the integration site selection of HIV-1 PICs when compared to the other A3 constructs may imply that deaminase-independent activity is an important factor, but not the sole factor, in influencing integration site selection. A key difference between the A3G [W94A] and A3G [W127A] mutants is that while A3G [W94A] can form homodimers, the A3G [W127A] mutant is less proficient in this regard^14^. Thus, the differences observed between A3G and A3G [W94A] may be due to a reduced affinity for nucleic acids, whereas the differences observed between A3G [W94A] and A3G [W127A] may be due to multimerization defects^14^.

Genomic positional effects have been shown to influence HIV-1 expression and latency reversal^54,69,70^. LADs represent a repressive chromatin environment tightly associated with the nuclear periphery^54,71^. SINEs (e.g., Alu repeats) and other transposed sequences are known to serve as direct silencers of gene expression due to their repressed chromatin marks (histone H3 methylated at Lys9)^72–74^. Moreover, some non-B DNA structures including G4, cruciform, triplex, and Z-DNA have been shown to potently silence expression of adjacent genes^75–85^. We showed here that both A3F and A3G increased the frequency of HIV-1 integration in or near LADs, SINEs and several non-B DNA motifs, and increased the proportion of latently infected cells in vitro, potentially implicating A3 in promoting integration in more transcriptionally silent regions of the genome. Interestingly, proviruses with deaminated 3’ LTR ends were highly enriched in and near gene silencing non-B DNA motifs compared to proviruses with non-deaminated 3’ LTR ends. However, it is currently unknown if these mutated viruses are replication-competent as mutations in the LTR are indicative that mutations may also be found elsewhere in the viral genome. Transcriptional activation and specific recovery of latent viral particles is a very complicated challenge as these constitute a very minor subset of the total pool of released viruses.

In conclusion, we have shown for the first time that A3 enzymes can modulate the integration site profile of HIV-1 via both deaminase-dependent and -independent mechanisms. While the strongest restrictive feature of these A3 proteins was determined to be their deaminase activity, even non-restrictive mutants maintained both the ability to interact with integrase and modulate integration site selection of HIV-1. Currently, the overall impact of A3 on influencing the integration site profile of HIV-1 and disease progression is unclear. A3 may represent a last-ditch effort to direct the intasome away from genes and into more potentially transcriptionally silent regions of the genome to promote proviral silencing. Further efforts are required to dissect this phenomenon and determine the influence of A3 on proviral silencing.

## Methods

This research complies with all relevant ethical regulations. Ethical clearance was obtained from the IRBs at the JCRC and UHCMC/CWRU (EM-10-07 and 10-05-35).

### Cell lines and plasmids

Cell lines were maintained in complete media (10% FBS, 100 U/mL Penicillin, and 100 µg/mL Streptomycin). HEK 293 T cells (ATCC CRL-3216^TM^) were maintained in complete DMEM with high glucose. CEM-SS cells (NIH AIDS #776) were maintained in complete RPMI. Both cell stocks were maintained in a humidified 37 °C incubator with 5% CO_2_. NL4-3- ΔVif/ΔEnv-eGFP was developed through site-directed mutagenesis of the NL4-3 ΔEnv-eGFP, which was originally obtained from the NIH AIDS Reagents Program (N.A.R.P.) (Catalog #11100)^14^. NL4-3- ΔVif/ ΔEnv-eGFP was pseudotyped with Vesicular Stomatitis Virus-G protein (VSV-G) (pMDG) as previously described^21,86^. pcDNA 3.1 (Invitrogen) was used as an empty vector control for transfection and all A3 expressing plasmids have been described previously^21,86^. Plasmids pHIV_GKO_ and pHIV_GKO-ΔU3LTR_ were kindly provided by Dr. Eric Verdin (Buck Institute).

### Virus production and infection

HEK 293 T cells were seeded at 7.5 × 10^5^ cells in each well of a 6-well plate. Twenty-four hours post-seeding, the cells were co-transfected with plasmids carrying the NL4-3-ΔEnv/ΔVif/eGFP reporter vector and pMDG, together with either empty vector or A3 plasmids as indicated using polyethylenimine (PEI)^87^. While the ratios of the NL4-3-ΔEnv/ΔVif/eGFP and pMDG plasmids remained constant (750 ng: 250 ng), the levels of co-transfected empty vector or A3 plasmid varied according to the experiment (30, 100, and 250 ng). For A3G and A3G-E259A, 20 ng were transfected to ensure sufficient cell numbers were available for flow cytometry analysis. The total amount of DNA transfected was kept constant using empty vector (pcDNA 3.1). Cells were incubated for 72 h to produce virus. Virus production was confirmed using western blotting with anti-p24Capsid (N.A.R.P. #1513), anti-FLAG (Clone M2; Sigma), and anti-β-Tubulin (ab21058; Abcam). Virus supernatants were collected, centrifuged at 500 × *g* for 5 min and filtered using a 450 nm syringe-filter to remove cellular debris. At this point, a Sandwich-ELISA was performed to determine levels of capsid protein (p24CA) using antibody isolated from Hybridoma 31-90-25 (#HB-9725; ATCC) and 183-H12-5C (N.A.R.P. #1513). Twenty hours after virus collection, CEM-SS cells were seeded in a 12-well plate at a density of 5 × 10^5^ cells per well and infected with normalized capsid levels (500, 100, or 20 ng) by spinoculation for 1 h at 900 × *g* without polybrene. Cells were incubated for 48 h and collected for downstream flow cytometry analysis and gDNA extraction. Flow Cytometry analysis was done by BD FACSCelesta using BD FACSDiva (Software v8.0.1)). Post-acquisition analysis was performed on a separate computer using FlowJo (software v10.4.2). Wizard gDNA Purification Kit (Promega) was used to isolate and purify gDNA from CEM-SS cells. Pseudotyped HIV_GKO_ and HIV_GKO-ΔU3LTR_ viruses were generated by co-transfecting HEK293T cells with pHIV_GKO_ or pHIV_GKO-ΔU3LTR_ and pMD.G in the presence or absence of plasmids carrying A3 (200 ng) for 72 h. Sandwich-ELISA was performed to determine levels of capsid protein (p24CA) as described above. CEM-SS cells were infected with HIV_GKO_ or pHIV_GKO-ΔU3LTR_ (equivalent of 120 ng p24CA protein) via spinoculation at 900 × *g* at room temperature for 1 h in the presence of 10 µg/ml polybrene (Sigma–Aldrich, #H9268-5G). Following spinoculation, virus was removed, and fresh medium was added to the well for 4 days. Infected cells were then run and analyzed by flow cytometry (BD FACSCelesta using BD FACSDiva (Software v8.0.1)). Post-acquisition analysis was performed on a separate computer using FlowJo (software v10.4.2). The proportions of latent or productive integrations were calculated by dividing the percentage of (csGFP−,mKO2+) or (csGFP+, mKO2+) cells, respectively, by the total percentage of positive cells.

### Quantification of integrated provirus using Alu-based qPCR

PowerUp^TM^ SYBR Master Mix (ThermoFisher) was used to quantify the relative levels of cells using a Viia^TM^7 Real-Time PCR Instrument (Applied Biosystems) with 50 ng gDNA template using the following primers: Actin-FWD 5’-CAT GTA CGT TGC TAT CCA GGC-3’ and Actin-REV 5’-CTC CTT AAT GTC ACG CAC GAT-3’. Cycling conditions: initial denaturation at 95 °C for 3 min, followed by 45 cycles of 95 °C for 15 s and 60 °C for 1 min. Data were analyzed using the QuantStudio (version 1.6.1) software. Next, similarly to a previously described protocol, Alu-PCR was performed using 50 ng of gDNA and PrimeStar GXL DNA Polymerase (Takara) using the following conditions: initial denaturation at 94 °C for 1 min followed by 30 cycles of 98 °C for 10 s, 55 °C for 15 s, and 68 °C for 10 min, ending with an additional extension step of 68 °C for 10 min^46^. All primers targeting the HIV-1 sequence were designed to exclude A3 dinucleotide hotspots to avoid inducing PCR biases. To quantify integrated eGFP sequences, the following primers were used: Alu1 5’-TCC CAG CTA CTG GGG AGG CTG AGG-3’, Alu2 5’-GCC TCC CAA AGT GCT GGG ATT ACA G-3’ and Lambda-eGFP-FWD 5’-ATG CCA CGT AAG CGA AAC TGT ACA ACT ACA ACA GCC ACA ACG TCT ATA TC-3’. A dilution of this was analyzed by ddPCR using the QX200 system (BioRad) with the following conditions: initial denaturation at 95 °C for 10 min followed by 45 cycles of 94 °C for 30 s, 60 °C for 30 s, and 72 °C for 30 s. This was followed by a final denaturation of 98 °C for 10 min. The following primers were used: LambdaE-F2 5’-ATG CCA CGT AAG CGA AAC TGT ACA ACT AC-3’, HIV eGFP REV 5’-TGA GGA TTG CTT AAA GAT TAT TGT TTT ATT ATT T-3’. This probe was used: /5HEX/ CCC CGT GCT /ZEN/ GCT GCC CRA CAA CCA CTA CC /3IABkFQ. To quantify integrated 5’ LTR sequences, the following primers were used: Lambda-R-U5-REV1 5’-AGT TTC GCT TAC GTG GCA TCA GAC GGG CAC ACA CTA CTT TGA GCA C-3’, Alu1 Comp 5’-CCT CAG CCT CCC CAG TAG CTG GGA-3’ and Alu2 Comp 5’-CTGT AAT CCC AGC ACT TTG GGA GGC-3’. A dilution of this was analyzed by ddPCR as described using the same conditions described above and the following primers: LambdaR-REV2 5’-GTT TCG CTT ACG TGG CAT CAG ACG G-3’ and Late U3-FWD 5’-GCT ACA TAT AAG CAG CTG CTT TTT GCC TGT AC −3’. The following probe was used: /5YAkYel/ CTT TAT TGA GGC T + T AAG + C + AG + T + G + GG T/3IABkFQ. Nucleotides followed by a+ (N+) indicate an LNA base to improve the melting temperature of the probe. Results from the Alu-PCR that quantified integrated proviruses using the eGFP sequence or the 5’ LTR sequence primers were then averaged.

### Immunoprecipitation

HEK 293 T cells were transfected with NL4-3-ΔEnv/ΔVif/eGFP and VSV-G and the viral supernatant was cleared of cellular debris. Virus supernatant was concentrated by ultracentrifugation through a 20% sucrose cushion at 100,000 × *g* for 3 h at 4 °C using a Type 70Ti. The viral pellet was resuspended in an isotonic 1% Triton-X 100 lysis buffer. At the same time, the virus producer cells were lysed using a soft lysis buffer with 500 mM NaCl to efficiently burst the nucleus and maximally release integrase. Protease inhibitors (Roche) were used at all times to prevent protein degradation. Salt-Active Nuclease (Sigma) was used to remove the gDNA according to the manufacturer’s protocol. Remaining cellular debris was removed by centrifugation at 4 °C at 17,000 × *g* for 10 min. Cellular and supernatant lysates were mixed together to maximize levels of viral components isolated. Overall salt levels were brought back to an isotonic state using sterile water. The aforementioned A3 or pcDNA 3.1 plasmids were each transfected individually in their own well at the same time as the NL4-3-ΔEnv/ΔVif/eGFP transfection. Seventy-two hours post-transfection, the cells were collected and lysed using an isotonic 1% Triton-X 100 lysis buffer. Lysates were sonicated to improve protein solubilization and centrifuged at 17,000 × *g* for 10 min at 4 °C to remove remaining cellular debris. A sample of each lysate was collected prior to immunoprecipitation to assess input levels. The viral lysate was equally divided among the cellular lysates containing A3 or controls. These lysates were then mixed with 30 µL of anti-FLAG conjugated magnetic µbeads (Miltenyi) and incubated on a tube rotator for 3 h at 4 °C. The µbeads were then magnetically isolated using a µcolumn according to manufacturer’s instructions. Samples were denatured and analyzed by Western blotting using anti-IN (IN-2, Santa Cruiz BioTechnology, sc-69721), anti-p24 Capsid (N.A.R.P. #1513), anti-FLAG (Clone M2; Sigma), anti-β-Tubulin (ab21058; Abcam). Western blots images were captured using Image Quant LAS4000 and analyzed using Image Quant TL software version 8.1.

### Integration site library and computational analysis

Genomic DNA was processed for integration site analysis and sequenced using the Illumina MiSeq platform^36,50^. Briefly, genomic DNA was restriction enzyme digested using MseI and NarI and the 3’ LTR-host genome junctions were amplified by ligation-mediated PCR. After gel purification of the PCR products, the purified DNA samples were processed using the Nextera XT DNA Sample Preparation kit. A limited-cycle PCR reaction was performed to amplify the insert DNA, which was then sequenced using Illumina MiSeq using 2×150 bp chemistry at the London Regional Genomics Centre (Robarts Research Institute, Western University, Canada). Fastq sequencing reads were quality trimmed and unique integration sites identified using our in-house bioinformatics pipeline^36^, which is called the Barr Lab Integration Site Identification Pipeline (BLISIP version 2.9) and includes the following updates: bedtools (v2.25.0), bioawk (awk version 20110810), bowtie2 (version 2.3.4.1), and restrSiteUtils (v1.2.9). HIV-1 3’ LTR-containing fastq sequences were identified and filtered by allowing up to a maximum of five mismatches with the reference NL4-3 3’ LTR sequence and if the 3’ LTR sequence had no match with any region of the human genome (GRCh37/hg19). Integration sites were determined from the sequence junction of the 3’ LTR and human genome sequences. All genomic sites in each dataset that hosted two or more sites (i.e., identical sites) were collapsed into one unique site for our analysis. Sites located in various common genomic features and non-B DNA motifs were quantified and heatmaps were generated using our in-house python program BLISIP Heatmap (BLISIPHA v1.0). Sites that could not be unambiguously mapped to a single region in the genome were excluded from the study. All non-B DNA motifs were defined according to previously established criteria^88^. Matched random control integration sites were generated by matching each experimentally determined site with 10 random sites in silico that were constructed to be the same number of bases away from the restriction site as was the experimental site^36^. Unique HIV 3’ LTRs were identified with BLISIP, aligned with MUSCLE (version 10.1.7)^89^ and gap-stripped with trimAl (version 1.2)^90^. All columns with gaps in more than 40% of the population were gap-stripped. Unique LTR sequence logos were generated using WebLogo (version 3.6)^52^.

### Patient sample collection and preparation

Samples were collected from the WHO, CAP, and NIH-VQA-accredited Center For AIDS Research (CFAR) Laboratory of the Joint Clinical Research Center (JCRC) in Kampala, Uganda. The JCRC is one of the first HIV treatment centers in the country to roll out ART and currently the only site licensed to provide INSTIs in the country. HIV-negative women of child-bearing age (18–35 years old) were recruited, volunteered (without compensation) as participants after counseling and signing a consent from ~2002 to 2007 in the Risk of HIV-1 Acquisition Study with Hormonal Contraceptive based on various inclusion and exclusion criteria. If a woman was diagnosed with HIV-1 during the parent study above, there was invitation to participate in an ancillary study to determine markers of disease progression, again under consent and following counseling. The patient database in the CFAR laboratory was used to access HIV-1 infected patient sample ID numbers only. Authors were blinded to all clinical data except for HIV-1 infection status. A total of 93 previously frozen and banked PBMC samples from HIV-1 infected patients receiving routine treatment care at the JCRC some of which also came from the Hormonal Contraception and HIV-1 Genital Shedding and Disease Progression among Women with Primary HIV Infection (GS) study were randomly collected^91^. Ethical clearance was obtained from the IRBs at the JCRC and UHCMC/CWRU (EM-10-07 and 10-05-35). Genomic DNA was extracted using DNeasy Blood & Tissue Kits (Qiagen) following manufacturer’s instructions and extracted DNA was stored at −80 °C.

### Statistics and reproducibility

All statistical tests were performed as described in figure legends using GraphPad Prism 9 version 9.4.1. No statistical method was used to predetermine sample size. No data were excluded from the analyses. The experiments were not randomized. The investigators were not blinded to allocation during experiments and outcome assessment. The investigators were blinded to all patient samples provided for integration site and LTR sequence analyses.

### Reporting summary

Further information on research design is available in the Nature Portfolio Reporting Summary linked to this article.

## Supplementary information

Supplementary Information

Description of Additional Supplementary Files

Supplementary Data File 1

Supplementary Data File 2

Supplementary Data File 3

Supplementary Data File 4

Supplementary Data File 5

Supplementary Data File 6

Supplementary Data File 7

Supplementary Data File 8

Supplementary Data File 9

Reporting Summary

## Data Availability

The source data generated in this study are provided in the Supplementary Information/Source Data files. Integration site locations in the human genome were obtained from the GRCh37/hg19 database (https://hgdownload.soe.ucsc.edu/downloads.html). The integration site sequencing data generated in this study have been deposited in the NCBI SRA database under accession codes SAMN31866157-SAMN31866258 [http://www.ncbi.nlm.nih.gov/bioproject/905178]. Source data are provided with this paper.

## Source data

Source Data

## Code availability

Bedtools (v2.25.0) (https://github.com/arq5x/bedtools2/releases), bioawk (awk version 20110810) (https://github.com/lh3/bioawk), bowtie2 (version 2.3.4.1) (https://github.com/BenLangmead/bowtie2), and restrSiteUtils (v1.2.9) (https://github.com/chasberry/integration-site-MRCs) were used to collect and analyze genomic DNA sequences and features. Integration sites in non-B DNA motifs were identified using the gquad R package (version 2.1-1), which is freely available as a standalone software package from the Comprehensive R Archive Network (https://cran.r-project.org/web/packages/gquad/index.html). This package provides functions for predicting non-B DNA. The key characteristics of the code and details pertaining to the test dataset can be found in the gquad documentation.
