# Supplementary material for: Antiretroviral APOBEC3 cytidine deaminases alter HIV-1 provirus integration site profiles
Source: Nat Commun. 2023 Jan 10;14:16. doi: 10.1038/s41467-022-35379-y (PMC9832166; doi:10.1038/s41467-022-35379-y)
Supplement: Supplementary file 1 — Supplementary Information [file 41467_2022_35379_MOESM1_ESM.pdf]

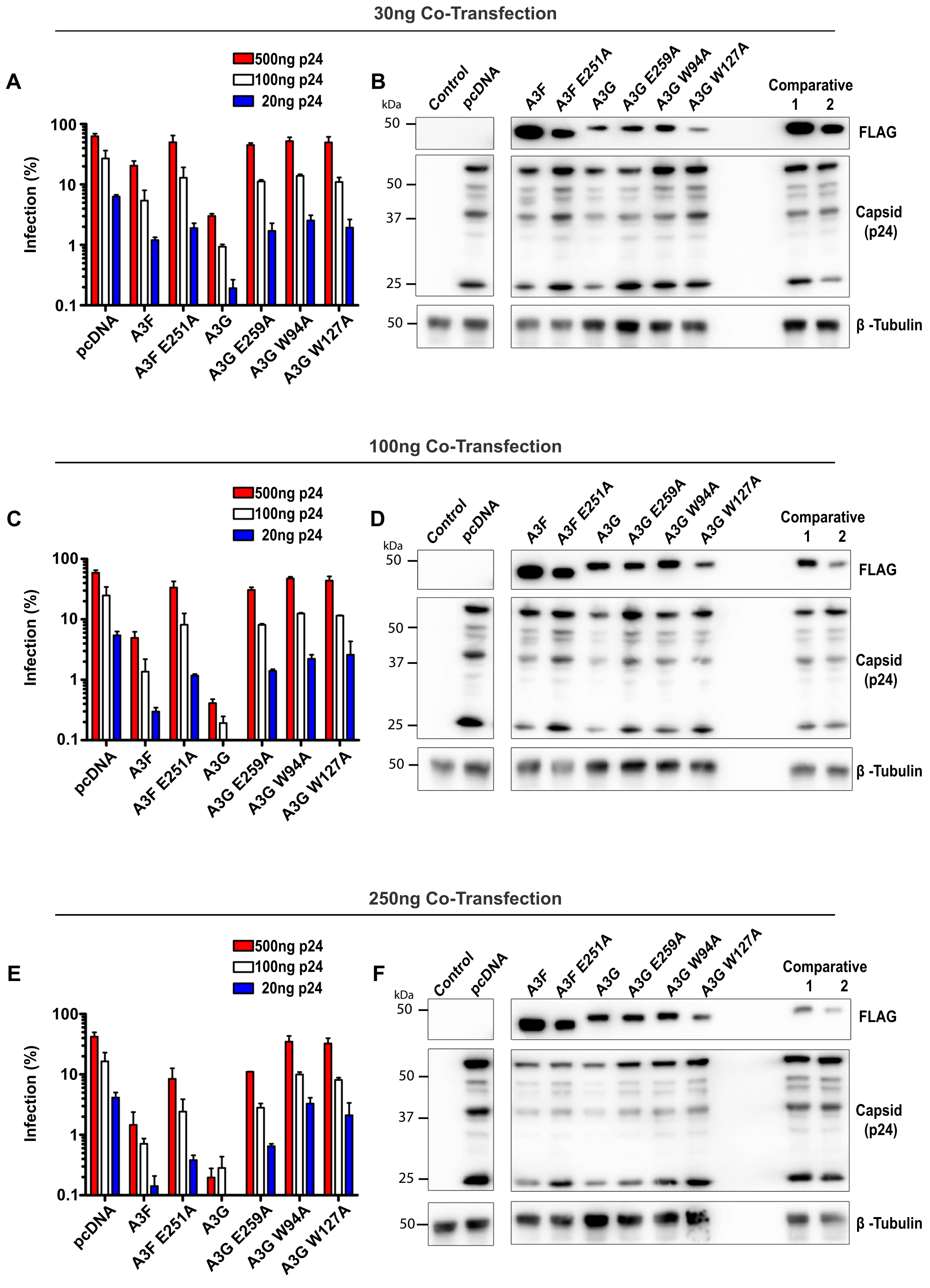

Figure S1: A3-mediated restriction of HIV-1 infection is directly related to levels of A3 proteins in viral producer cells. CEM-SS cells were infected with pre-determined levels of capsid (p24) protein (20 ng, 100 ng or 500 ng) as measured by ELISA. The percentage of infected cells was determined by flow cytometry. Productively infected cells express the eGFP reporter gene encoded in the viral genome. Data are presented as mean values +/- SD (A, C and E). Production of intracellular viral proteins was analyzed by Western blotting of transfected virus producer cells using anti-FLAG, anti-p24CA or anti-β tubulin (B, D and F). Each panel is representative of virus produced from cells transfected with 30 ng (A and B), 100 ng (C and D) or 250 ng (E and F) of A3 plasmid. Data shown are representative of three independent experiments. Source data are provided as a Source Data file.

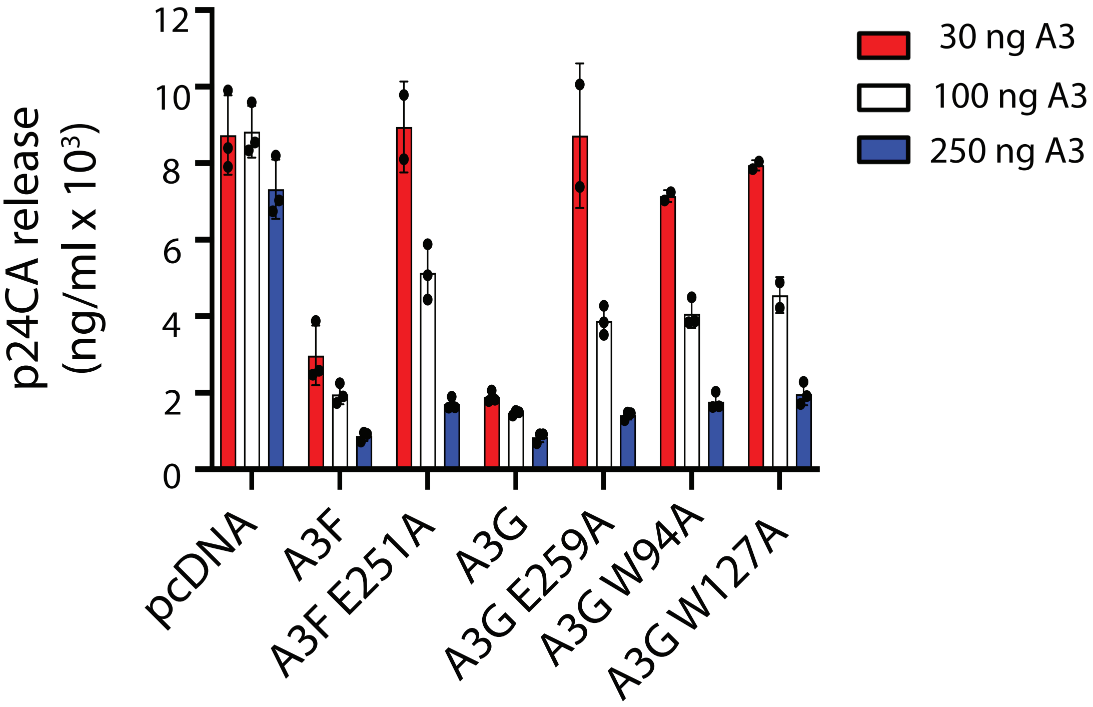

Figure S2: Measurements of HIV-1 particle release. A. Particle release was measured by p24CA sandwich ELISA for all transfection conditions (i.e., 30ng, 100ng or 250ng of co-transfected A3 or pcDNA plasmid) to produce virus. Data are presented as mean values +/- SD. Data shown are representative of three independent experiments. Source data are provided as a Source Data file.

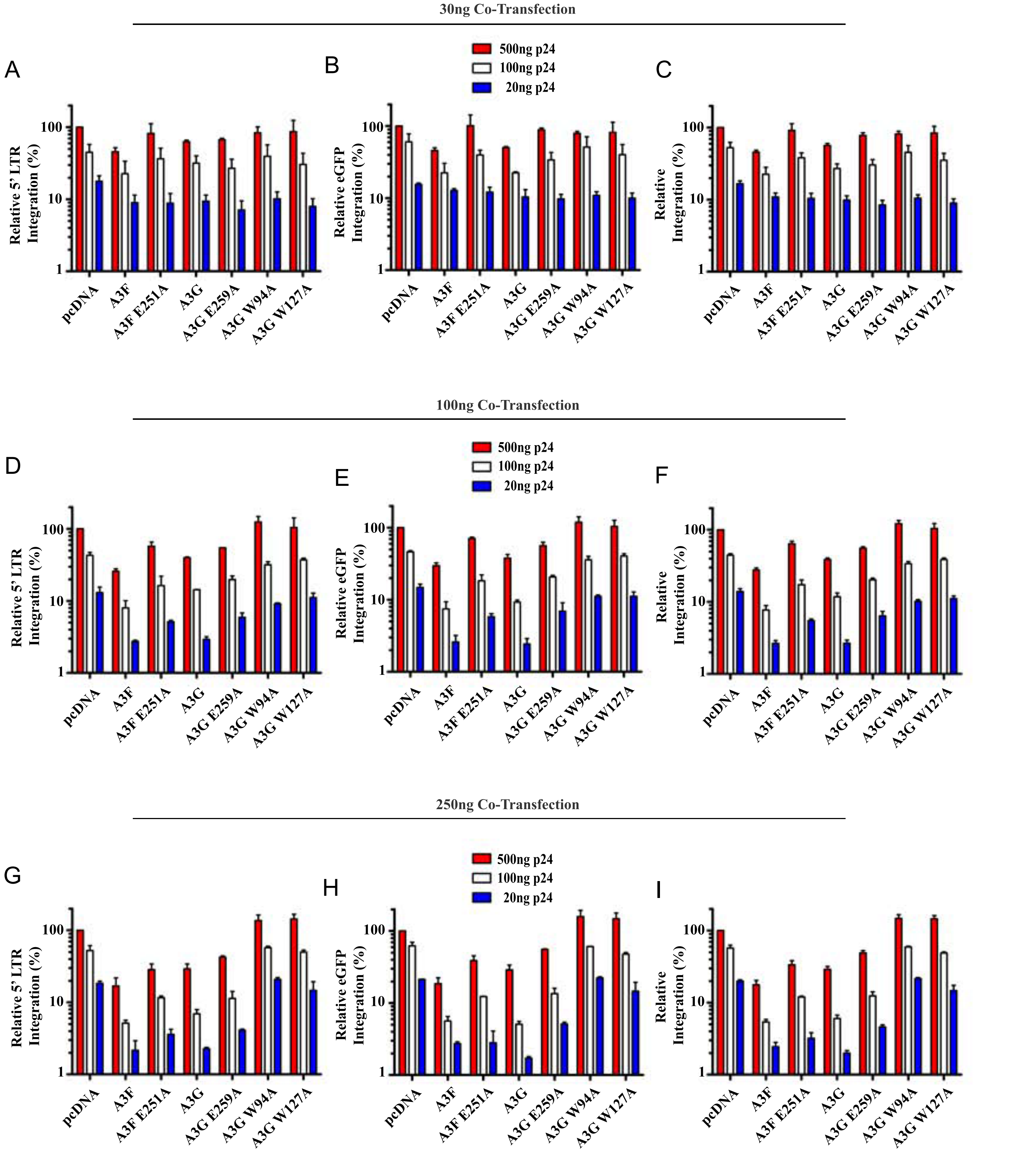

Figure S3: Restriction of HIV-1 integration is directly related to levels of A3 proteins in viral producer cells. The level of integrated provirus in infected CEM-SS cells (as described in Figure S1) was determined by Alu-based ddPCR targeting either the 5' LTR (A, D and G) or the eGFP reporter gene (B, E and H). Levels of proviral targets were normalized to a control gene (beta-actin) by qPCR. Similar results were obtained for either integration analysis methods, with the average quantification of the two shown in panels C, F and I. Each panel is representative of the integration levels from cells transfected with 30 ng (A-C), 100 ng (D-F) and 250 ng (G-I) of A3 plasmid. Data are presented as mean values +/- SD. Data shown are representative of three independent experiments. Source data are provided as a Source Data file.

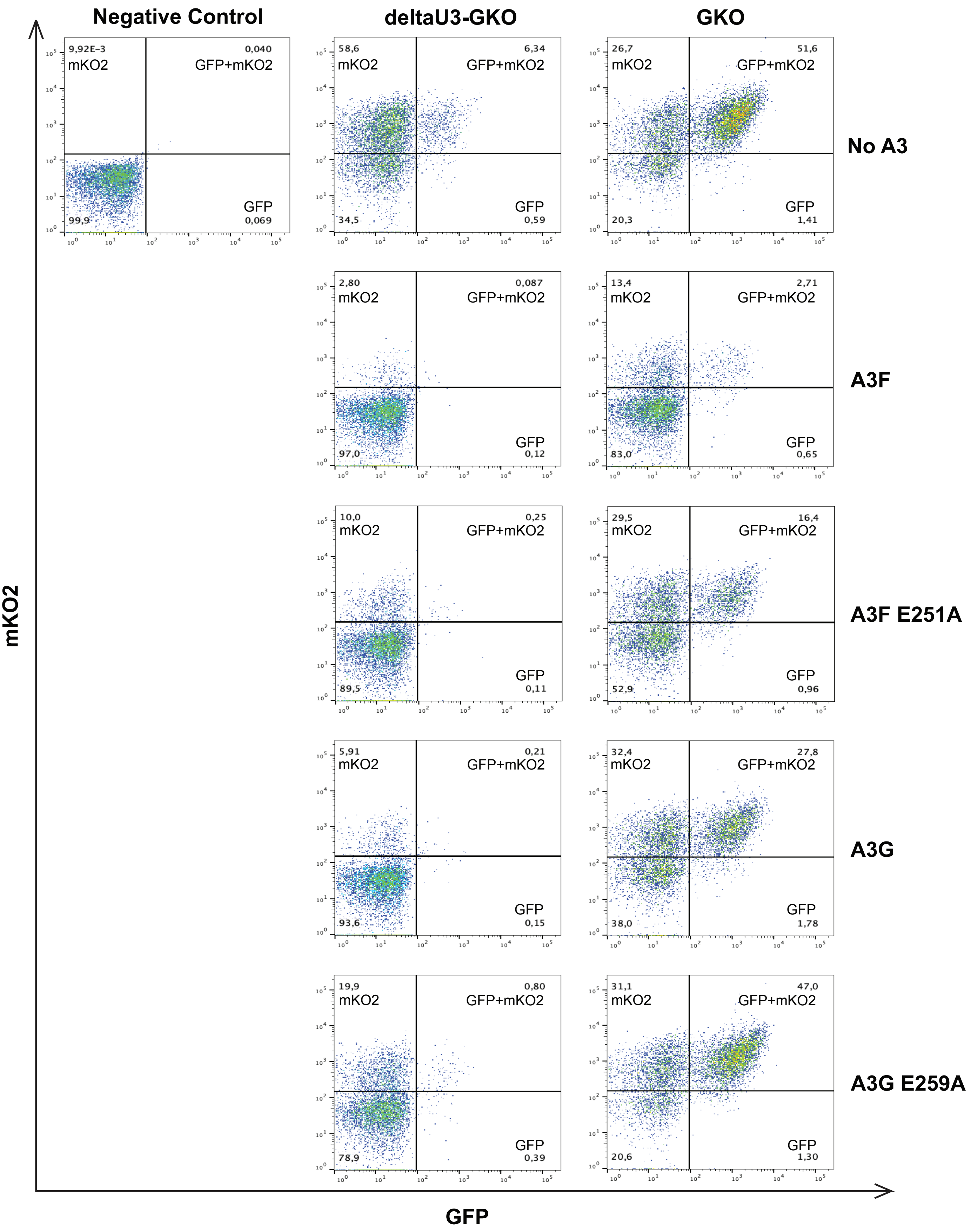

Figure S4: Flow cytometry analysis of reporter gene expression of integrated GKO/ deltaU3-GKO virus. Analyses were performed on infected CEM-SS cells. X-axis represents GFP expression, and the Y-axis represents mKO2 expression. The percentage of each cell population is indicated in the quadrant. One representative experiment of two is shown. Source data are provided as a Source Data file.

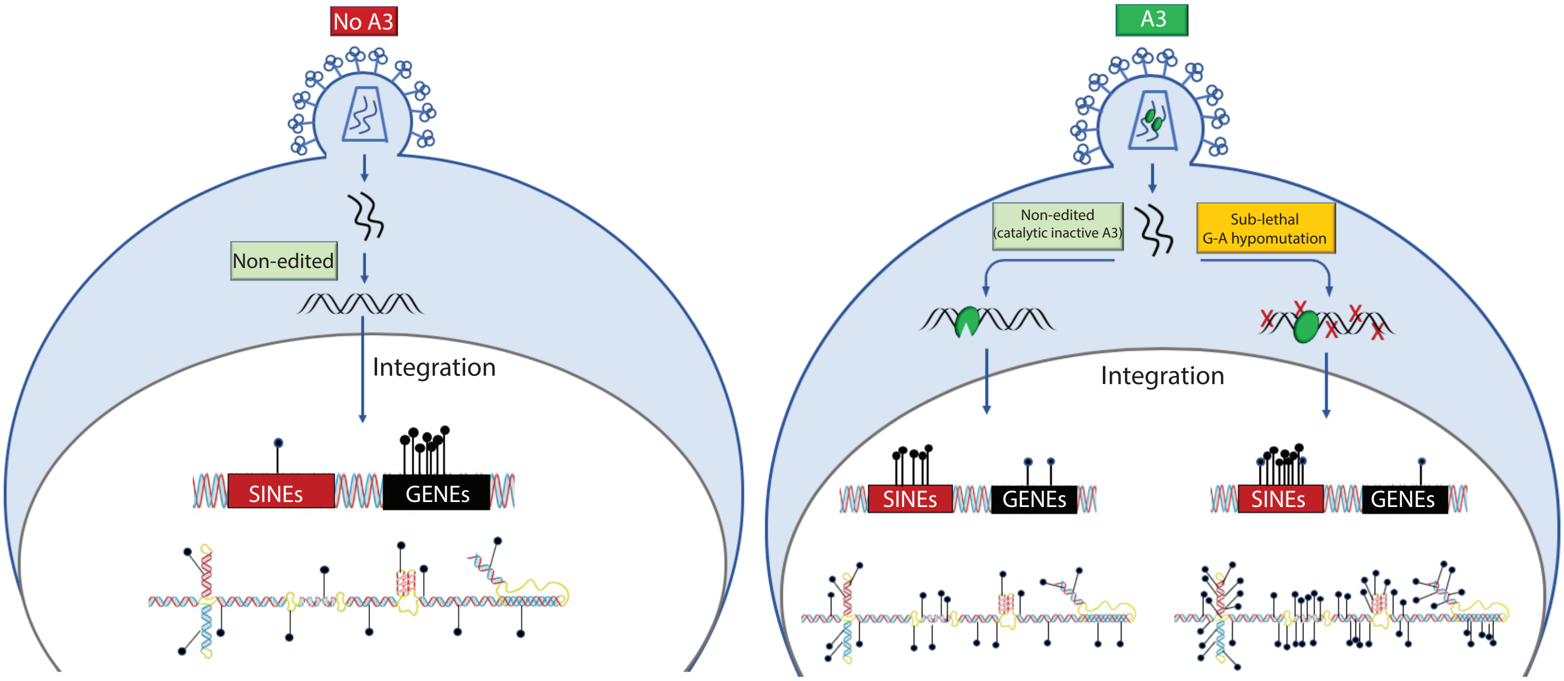

Figure S5: Schematic depicting the influence of APOBEC3 (A3) proteins on HIV integration site targeting. Left, in the absence of A3, HIV has a strong preference for integrating into genes. Right, both catalytic active and non-catalytic A3 mutants decrease integration into genes and increase integration into SINE elements and in transcription-silencing non-B DNA features.
