# Supplementary material for: Antiretroviral APOBEC3 cytidine deaminases alter HIV-1 provirus integration site profiles
Source: Nat Commun. 2023 Jan 10;14:16. doi: 10.1038/s41467-022-35379-y (PMC9832166; doi:10.1038/s41467-022-35379-y)
Supplement: Supplementary file 2 — Description of Additional Supplementary Files [file 41467_2022_35379_MOESM2_ESM.pdf]

Supplementary Data Legends

Supplementary Data File 1: Integration site distribution in common genomic features from infected cells expressing various APOBEC3 constructs.

Supplementary Data File 2: Integration site heatmaps with fold changes in integration site abundance in and near common genomic DNA features from infected cells expressing various APOBEC3 constructs.

Supplementary Data File 3: Integration site distribution from HIV-1-infected CEM-SS cells expressing increasing concentrations of APOBEC3 or APOBEC3 mutant proteins.

Supplementary Data File 4: Integration site distribution in and near non-B DNA features from infected cells expressing various APOBEC3 constructs.

Supplementary Data File 5: Integration site heatmaps with fold changes in integration site abundance in and near non-B DNA features from infected cells expressing various APOBEC3 constructs.

Supplementary Data File 6: Effect of APOBEC3 expression on the number of integration hotspots.

Supplementary Data File 7: Integration site distribution in and near common genomic DNA and non-B DNA features of A3F-mutated LTRs.

Supplementary Data File 8: Integration site distribution in and near common genomic DNA and non-B DNA features of A3G-mutated LTRs.

Supplementary Data File 9: Integration site distribution in and near common genomic DNA and non-B DNA features of A3G-mutated LTRs in vivo.
