# Supplementary material for: Antiretroviral APOBEC3 cytidine deaminases alter HIV-1 provirus integration site profiles
Source: Nat Commun. 2023 Jan 10;14:16. doi: 10.1038/s41467-022-35379-y (PMC9832166; doi:10.1038/s41467-022-35379-y)
Supplement: Supplementary file 12 — Reporting Summary [file 41467_2022_35379_MOESM12_ESM.pdf]

nature portfolio

Corresponding author(s): Marc-Andre Langlois and Stephen D. Barr

Last updated by author(s): Nov 23, 2023

Reporting Summary

Nature Portfolio wishes to improve the reproducibility of the work that we publish. This form provides structure for consistency and transparency in reporting. For further information on Nature Portfolio policies, see our Editorial Policies and the Editorial Policy Checklist.

Statistics

For all statistical analyses, confirm that the following items are present in the figure legend, table legend, main text, or Methods section.

n/a	Confirmed	
☐	☒	The exact sample size (n) for each experimental group/condition, given as a discrete number and unit of measurement
☐	☒	A statement on whether measurements were taken from distinct samples or whether the same sample was measured repeatedly
☐	☒	The statistical test(s) used AND whether they are one- or two-sided<br>*Only common tests should be described solely by name; describe more complex techniques in the Methods section.*
☐	☒	A description of all covariates tested
☐	☒	A description of any assumptions or corrections, such as tests of normality and adjustment for multiple comparisons
☐	☒	A full description of the statistical parameters including central tendency (e.g. means) or other basic estimates (e.g. regression coefficient) AND variation (e.g. standard deviation) or associated estimates of uncertainty (e.g. confidence intervals)
☐	☒	For null hypothesis testing, the test statistic (e.g. F, t, r) with confidence intervals, effect sizes, degrees of freedom and P value noted<br>*Give P values as exact values whenever suitable.*
☒	☐	For Bayesian analysis, information on the choice of priors and Markov chain Monte Carlo settings
☒	☐	For hierarchical and complex designs, identification of the appropriate level for tests and full reporting of outcomes
☐	☒	Estimates of effect sizes (e.g. Cohen's d, Pearson's r), indicating how they were calculated

Our web collection on statistics for biologists contains articles on many of the points above.

Software and code

Policy information about availability of computer code

Data collection	Bedtools (v2.25.0), bioawk (awk version 20110810), bowtie2 (version 2.3.4.1), and restrSiteUtils (v1.2.9) were used to collect and analyse genomic DNA sequences and features, MUSCLE (MEGAX version 10.1.7), TrimAl (version 1.2), WebLogo (version 3.6), FACSDiva (software v8.0.1), Quantstudio (version 1.6.1).
Data analysis	Integration sites in non-B DNA motifs were identified using gquad R package (version 2.1-1; https://cran.r-project.org/web/packages/gquad/index.html); FlowJo (v10.4.2)

For manuscripts utilizing custom algorithms or software that are central to the research but not yet described in published literature, software must be made available to editors and reviewers. We strongly encourage code deposition in a community repository (e.g. GitHub). See the Nature Portfolio guidelines for submitting code & software for further information.

Data

Policy information about availability of data

All manuscripts must include a data availability statement. This statement should provide the following information, where applicable:

- Accession codes, unique identifiers, or web links for publicly available datasets
- A description of any restrictions on data availability
- For clinical datasets or third party data, please ensure that the statement adheres to our policy

Data Availability

Integration site locations in the human genome were obtained from the GRCh37/hg19 database (https://hgdownload.soe.ucsc.edu/downloads.html). Source data are provided with this paper. The integration site sequencing data generated in this study have been deposited in the NCBI SRA database under accession codes SAMN31866157-SAMN31866258 [http://www.ncbi.nlm.nih.gov/bioproject/905178]. The source data generated in this study are provided in the Supplementary Information/Source Data file.

Code Availability
Bedtools (v2.25.0) (https://github.com/arq5x/bedtools2/releases), bioawk (awk version 20110810) (https://github.com/lh3/bioawk), bowtie2 (version 2.3.4.1) (https://github.com/BenLangmead/bowtie2), and restrSiteUtils (v1.2.9) (https://github.com/chasberry/integration-site-MRCs) were used to collect and analyse genomic DNA sequences and features. Integration sites in non-B DNA motifs were identified using the gquad R package (version 2.1-1) which is freely available as a standalone software package from the Comprehensive R Archive Network (https://cran.r-project.org/web/packages/gquad/index.html). This package provides functions for predicting non-B DNA. The key characteristics of the code and details pertaining to the test dataset can be found in the gquad documentation.

Human research participants

Policy information about studies involving human research participants and Sex and Gender in Research.

Reporting on sex and gender	The parent study that had recruited participants was examining the risk of HIV-1 acquisition in women on hormonal contraceptives. Only women that self reported their sex were enrolled for this study after screening and informed consent. Not relevant to this study but all women underwent gynecological exam as part of standard of care and for the studies. Samples were obtained for future HIV research studies related to viral fitness that may impact disease progression in these women which pertains to this study. There was are also HIV-1 infected patients who are receiving antiretroviral treatment and tested for drug resistance as standard of care for which sex and gender is not screening tool or considered for normal patient care at the Joint Clinical Research Center.
Population characteristics	None of the co-variate analyses or sociodemographic information was necessary to report in this study. Aside from Dr. Eric Arts, the other authors of this study were blinded to all clinical and participant information in the women enrolled in the clinical study. None of the authors including Dr. Arts has any patient identifiers or clinical information on the samples obtained for routine drug resistance testing as standard of care.
Recruitment	Samples were collected from the WHO, CAP, and NIH-VQA-accredited Center For AIDS Research (CFAR) Laboratory of the Joint Clinical Research Center (JCRC) in Kampala, Uganda. The JCRC is one of the first HIV treatment centers in the country to roll out ART and currently the only site licensed to provide INSTIs in the country. HIV-negative women of child-bearing age (18-35 years old) were recruited, volunteered (without compensation) as participants after counseling and signing a consent from approximately 2002 to 2007 in the Risk of HIV-1 Acquisition Study with Hormonal Contraceptive based on various inclusion and exclusion criteria. If a woman was diagnosed with HIV-1 during the parent study above, there was invitation to participate in an ancillary study to determine markers of disease progression, again under consent and following counseling. The patient database in the CFAR laboratory was used to access HIV-1 infected patient sample ID numbers only. A total of 93 previously frozen and banked PBMC samples from HIV-1 infected patients receiving routine treatment care at the JCRC some of which also came from the Hormonal Contraception and HIV-1 Genital Shedding and Disease Progression among Women with Primary HIV Infection (GS) study were randomly collected.
Ethics oversight	Ethical clearance was obtained from the IRBs at the JCRC and UHCMC/CWRU (EM-10-<br>579 07 and 10-05-35)

Note that full information on the approval of the study protocol must also be provided in the manuscript.

Field-specific reporting

Please select the one below that is the best fit for your research. If you are not sure, read the appropriate sections before making your selection.

☒ Life sciences ☐ Behavioural & social sciences ☐ Ecological, evolutionary & environmental sciences

For a reference copy of the document with all sections, see nature.com/documents/nr-reporting-summary-flat.pdf

Life sciences study design

All studies must disclose on these points even when the disclosure is negative.

Sample size	A total of 93 whole blood samples were collected from patients receiving routine treatment care at the JCRC in Uganda. We randomly selected as many Ugandan participants as possible in order to maximize the number of integration sites for analysis. Given that each participant hosted dozens of integrations sites, we were able to achieve robust statistical analysis from the 93 participants.
Data exclusions	In our flow cytometry experiments with the dual-color HIV vector, we excluded cells not expressing either marker (csGFP-,mKO2-) which comprise uninfected or dead cells, cells containing defective proviruses, and/or cells containing proviruses latent for both csGFP and mKO2 expression. We were interested in analyzing latently infected cells but because we could not differentiate cells latent for csGFP and mKO2 expression from uninfected cells or cells containing defective proviruses from this particular population of sorted cells, they were excluded from analysis. This was predetermined before the experiment took place. In our analysis of LTR sequences, to ensure that we extracted sequences with sufficient homology with the HIV-1 LTR, LTR sequences containing more than 5 mismatches with the reference

	HIV-1 LTR sequence were filtered out and not included in the analysis. This was a predetermined quality control criteria.
Replication	All experimental infections were successfully replicated three independent times. We also performed infections at different concentrations of virus and different APOBEC3 concentrations (3 independent replicates) which all yielded results consistent with the conclusions of the study.
Randomization	The patient database in the CFAR laboratory was used to identify patient samples that would contain HIV-1 infected cells. The 93 Ugandan participant samples were randomly selected from the cohort without bias for clinical status. Allocation of samples into different experimental groups was not relevant in this study because all samples were grouped into one experimental group of infected patient DNA.
Blinding	Blinded DNA samples were processed and sequenced to determine integration site profiles.

Reporting for specific materials, systems and methods

We require information from authors about some types of materials, experimental systems and methods used in many studies. Here, indicate whether each material, system or method listed is relevant to your study. If you are not sure if a list item applies to your research, read the appropriate section before selecting a response.

Materials & experimental systems

n/a	Involved in the study
☐	☒ Antibodies
☐	☒ Eukaryotic cell lines
☒	☐ Palaeontology and archaeology
☒	☐ Animals and other organisms
☒	☐ Clinical data
☒	☐ Dual use research of concern

Methods

n/a	Involved in the study
☒	☐ ChIP-seq
☐	☒ Flow cytometry
☒	☐ MRI-based neuroimaging

Antibodies

Antibodies used

Antibodies used in Western blot and IP
1- Mouse anti-Human Immunodeficiency Virus 1 (HIV-1) p24 Monoclonal (183-H12-5C, NARP, #1513) dilution 1:2000 final concentration: 5ug/ml
2- Monoclonal anti-FLAG (Clone M2; Sigma, Cat# F1804-200ug), dilution 1:1500 final concentration 0.67ug/ml
3- HRP Rabbit polyclonal to β-Tubulin (ab21058; Abcam) dilution 1:1500 final concentration 0.25ug/ml
4- Monoclonal anti Human Immunodeficiency Virus 1 (HIV-1) Integrase (IN)-N2 (N.A.R.P) (cat#ARP-7375, lot#060884) 3G4 (1mg/ml) dilution used 1:750.

Antibodies used in sandwiched ELISA:
1- Mouse anti-p24 capsid 183-H12-5C (N.A.R.P. #1513) final concentration: 5ug/ml
2- Hybridoma 31-90-25 (#HB-9725; ATCC) used to generate primary antibody-biotin conjugated and used at a concentration: 10ug/ml

Validation

Mouse anti-p24 (183-H12-5C) is used as a capture antibody in ELISA and primary antibody in Western blots at a concentration of 5ug/ml. 31-90-25 p24 antibody is used as a primary antibody-biotin conjugated (10ug/ml). Mouse anti-FLAG is used at a concentration of (0.67ug/ml) and rabbit anti-beta-tubulin (0.25ug/ml).

Monoclonal anti-FLAG: The monoclonal antibody detects only the target protein band(s) on a Western blot from an E. coli, plant or mammalian crude cell lysate. The monoclonal antibody detects as little as 2 ng of target protein by dot blot. The Western blot is tested down to 10 ng. Validation: A Western blot was performed to demonstrate that the ANTI-FLAG M2, Affinity Purified antibody displays exquisite specificity for the epitope-tagged fusion protein. (https://www.sigmaaldrich.com/deepweb/assets/sigmaaldrich/product/documents/144/194/vol6_iss2_antiflag_m2.pdf.)
Monoclonal mouse anti-Human Immunodeficiency Virus 1 (HIV-1): ARP-3537 is a monoclonal antibody to HIV-1 p24 .This antibody was produced in cell culture and purified by Protein G chromatography. It originates from a hybridoma. The hybridoma was created by immunizing a Balb/c mouse and fusing the resulting splenocytes with SP2/0 myeloma cells. This antibody is cross reactive with HIV-2 p24 and SIV p27 (Wehrly, K., & Chesebro, B. (1997). p24 antigen capture assay for quantification of human immunodeficiency virus using readily available inexpensive reagents. Methods,12(4), 288-293. doi:10.1006/meth.1997.048)
HRP Rabbit polyclonal to β-Tubulin
This antibody is suitable for western blot. It gave a positive signal against in both Human brain tissue and Human tonsil tissue as well as the following whole cell lysates: MCF-7, Jurkat, Ramos, HeLa; NIH3T3; PC12. Predicted molecular weight 49kDa.
Monoclonal Anti-IN
ARP-7375 is a monoclonal antibody to HIV-1 HXB2 integrase. This antibody originates from a hybridoma. The hybridoma was created by immunizing Balb/c mice (females) with recombinant HIV-1 HXB2 integrase and fusing the resulting splenocytes with P3X63 Ag8.653 myeloma cells. Known applications are western blot, ELISA and immunofluorescence. Recombinant HIV-1 (HXB2) IN used as antigen. Reacts with the C-terminus aa 22-31and 82-101. Inhibits IN activity. Works by ELISA, Western blot and immunofluorescence; concentration should be titered (Nilsen BM, Haugan IR, Berg K, Olsen L, Brown PO, Helland DE. Monoclonal antibodies against human immunodeficiency virus type 1 integrase: epitope mapping and differential effects on integrase activities in vitro. J Virol 70:1580-1587, 1996).

Eukaryotic cell lines

Policy information about cell lines and Sex and Gender in Research

Cell line source(s)

HEK 293T cells (ATCC CRL-3216), CEM-SS cells (NIH AIDS #776). The Ugandan participants were female.

Authentication

None of the cell lines used were genetically authenticated. The cell lines were assessed morphologically.

Mycoplasma contamination

HEK 293T cells (ATCC CRL-3216), CEM-SS cells (NIH AIDS #776) were not tested for mycoplasma contamination.

Commonly misidentified lines
(See ICLAC register)

None

Flow Cytometry

Plots

Confirm that:

- [x] The axis labels state the marker and fluorochrome used (e.g. CD4-FITC).
- [x] The axis scales are clearly visible. Include numbers along axes only for bottom left plot of group (a 'group' is an analysis of identical markers).
- [x] All plots are contour plots with outliers or pseudocolor plots.
- [x] A numerical value for number of cells or percentage (with statistics) is provided.

Methodology

Sample preparation

CEM-SS cells (NIH AIDS #776) were infected with dual-color HIV pseudotyped virus. Seventy-two hours post infection, cells were picked up using 500 ul of 1X PBS+5mM EDTA. Cells were centrifuged at 500xg for 5 minutes and resuspended in 1ml of 1x PBS to wash. Cells were centrifuged again at 500xg for 5 mins and then resuspended in 600ul basic sort buffer (1X PBS, 5mM EDTA, 25mM HEPES, 1% globulin free bovine serum albumin, pH 7.2-7.4, 0.2um filtered) and fixed with the addition of 600ul 4% paraformaldehyde (PFA). Fixed cell solutions were then 40um filtered (Bio Basic Inc. #SP104151) into 5ml Falcon polystyrene round bottom FACS tubes (Corning #352054, Tewsbury) for flow cytometry analysis.

Instrument

Samples were run and analyzed on a BD FACSCelestra using BD FACSDiva (software v8.0.1)

Software

Post-acquisition analysis was performed on a separate computer using FlowJo (software v10.4.2)

Cell population abundance

Initial cell populations were selected through the forward and side scatter plots by excluding debris and dead cells (smallFSC and SSC) followed by FSC-A/FSC-H gating to select singlet cells. 10,000-20,000 events were originally collected from which positively-gated cells showed 90-95% purity.

Gating strategy

All samples were FSC-A and SSC-A gated, followed by FSC-A/FSC-H gating to select singlet cells. Uninfected control cells were used to set gates.

☒ Tick this box to confirm that a figure exemplifying the gating strategy is provided in the Supplementary Information.
